# Chlorambucil Conjugated Ugi Dendrimers with PAMAM-NH_2_ Core and Evaluation of Their Anticancer Activity

**DOI:** 10.3390/pharmaceutics11020059

**Published:** 2019-02-01

**Authors:** Nalin Seixas, Bruno B. Ravanello, Ibrahim Morgan, Goran N. Kaluđerović, Ludger A. Wessjohann

**Affiliations:** 1 Department of Bioorganic Chemistry, Leibniz Institute of Plant Biochemistry, Weinberg 3, 06120, Halle (Saale), Germany; Nalin.deSeixasBorges@ipb-halle.de (N.S.); bbrisoll@ipb-halle.de (B.B.R.); Ibrahim.Morgan@ipb-halle.de (I.M.); Goran.Kaluderovic@ipb-halle.de (G.N.K.); 2 Department of Engineering and Natural Sciences, University of Applied Sciences Merseburg, Eberhard-Leibnitz-Strasse 2, 06217 Merseburg, Germany

**Keywords:** cell type selective uptake, anticancer drugs, PAMAM-NH_2_ dendrimer, chlorambucil, Ugi reaction, non-cancerous mouse NIH3T3 fibroblasts, PC-3 prostate cancer cell, HT-29 colon cancer cell, multicomponent reaction

## Abstract

Herein, a new Ugi multicomponent reaction strategy is described to enhance activity and solubility of the chemotherapeutic drug chlorambucil through its conjugation to poly(amidoamine) (PAMAM-NH_2_) dendrimers with the simultaneous introduction of lipidic (*i*-Pr) and cationic (–NH_2_) or anionic (–COOH) groups. Standard viability assays were used to evaluate the anticancer potential of the water-soluble dendrimers against PC-3 prostate and HT-29 colon cancer cell lines, as well as non-cancerous mouse NIH3T3 fibroblasts. It could be demonstrated that the anticancer activity against PC-3 cells was considerably improved when both chlorambucil and –NH_2_ (cationic) groups were present on the dendrimer surface (**1b**). Additionally, this dendrimer showed activity only against the prostate cancer cells (PC-3), while it did not affect colon cancer cells and fibroblasts significantly. The cationic chlorambucil-dendrimer **1b** blocks PC-3 cells in the G2/M phase and induces caspase independent apoptosis.

## 1. Introduction

The chemotherapeutic drug chlorambucil (CLB) is a nitrogen mustard derivative used in the treatment of some types of cancer, e.g., Hodgkin and non-Hodgkin lymphoma, as well as chronic lymphocytic leukemia [1,2,3,4]. The anticancer activity of CLB is based on its alkylation properties that damages the DNA and interferes with its replication.

Despite being an FDA approved drug, chlorambucil is not water-soluble and has poor specificity towards cancer cells, which causes pronounced side effects, hence it has been replaced by other chemotherapeutic drugs, such as fludarabine [5,6,7]. Nowadays, macromolecules, such as polymers, dendrimers and inorganic nanoparticles, have been used as nanosized drug carriers for chemotherapeutic drugs in order to increase water solubility, enhance cancer cell affinity, and minimize toxicity [8,9,10,11,12,13,14].

Moreover, amphiphilic polyamines (polycations), such as the famous Tat-sequence, can facilitate cellular uptake of therapeutic molecules and enhance their efficiency [15,16,17,18]. The uptake as well as the specificity depend on the amount and positioning of the amino groups, and in the wrong combination can lead to detrimental reactions, like hemolysis [19,20,21], or in other cases, to preferential uptake, e.g., some prostate cells have a specific affinity to certain polyamine structures (spermine, spermidine, etc.) [22,23].

Previous studies of chlorambucil conjugated with dendrimers via amide or ester linkages demonstrated that the conjugates were more potent anticancer agents than CLB itself against both MCF-7 and MDA-MB-231 breast cancer cell lines. It was found that the conjugates inhibit the proliferation by increasing apoptotic and necrotic cells, i.e., cell death was higher than caused by CLB alone (not normalized to 1 equivalent of chlorambucil) [24,25]. Although many efforts have been made to analyze the effects of chlorambucil-dendrimer conjugates on cancer cells, no study so far has shown the effect on non-cancerous cells.

Recently, our group invented the first multicomponent-based dendrimers (e.g., by Ugi reaction) [26]. Based on this protocol, a new synthetic strategy for chimeras of classical and Ugi-dendrimers is presented here for the purpose of improving cancer cell affinity and water solubility of chlorambucil. The Ugi four-component reaction (U-4CR) is a multicomponent reaction (MCR), most commonly between an amine, an aldehyde, a carboxylic acid, and an isocyanide to afford a peptoid-like backbone [27,28,29]. The U-4CR allows the creation of a new generation on the dendrimers surface by Ugi reaction of poly(amidoamine) (PAMAM-NH_2_) dendrimer, introducing three new self-assembling moieties with distinct properties in a simple and efficient one-pot procedure. Herein, PAMAM-NH_2_ dendrimer generation 0 was functionalized with chlorambucil and with lipidic (*i*-Pr) and cationic (–NH_2_) or anionic (–COOH) groups by Ugi multicomponent reactions. The effect of the different surface groups was evaluated in the cytotoxic activity against HT-29 colon and PC-3 prostate cancer cell lines, as well as non-cancerous mouse NIH3T3 fibroblasts using MTT (3-(4,5-dimethylthiazol-2-yl)-2,5-diphenyltetrazolium bromide) and CV (crystal violet) viability assays.

## 2. Materials and Methods

### 2.1. Materials

PAMAM-NH_2_ dendrimer generation 0 (20 wt.% solution in methanol) was acquired from Sigma-Aldrich (Germany, manufactured by Dendritech, Midland, MI, USA). Chlorambucil was acquired from Alfa Aesar (Karlsruhe, Germany). HT-29 cells were provided by Professor B. Seliger (Immunology Department, Martin Luther University Halle-Wittenberg, Halle (Saale), Germany). PC-3 and NIH3T3 cell lines were purchased from German Collection of Microorganisms and Cell Cultures (Leibniz-DSMZ, Braunschweig, Germany). Fetal calf serum (FCS), RPMI-1640, phosphate-buffered saline (PBS), dimethyl sulfoxide (DMSO), 3-methyl adenine (3-MA), carboxyfluorescein diacetate succinimidyl ester (CFSE), 3-(4,5- dimethylthiazol-2-yl)-2,5-diphenyltetrazolium bromide (MTT), crystal violet (CV), 4′,6-diamidino-2-phenylindole (DAPI), ethylenediamine tetraacetic acid (EDTA), propidium iodide (PI), and 4-amino-5-methylamino-2′,7′-difluorescein (DAF-FM) were purchased from Sigma-Aldrich (St. Louis, MO, USA). Annexin V-FITC (AnnV) was obtained from Santa Cruz Biotechnology (Dallas, TX, USA). Acridine orange (AO) was from Labo-Moderna (Paris, France). All other chemicals and reagents purchased from commercial source were obtained from Sigma-Aldrich (Taufkirchen, Germany) or Alfa Aesar (Karlsruhe, Germany), and were used without further purification.

### 2.2. Analytical Methods

Merck silica gel 60 (0.040–0.063 mm) was used for flash column chromatography (approximately 35 g of silica gel/1g of crude product). The ^1^H NMR and ^13^C NMR spectra (at 25 °C) were recorded in MeOD as solvent on an Agilent DD2 400 spectrometer (Waldbronn, Germany) at 400 MHz and 101 MHz, respectively. Reported ^1^H and ^13^C NMR chemical shifts (δ; in ppm) are relative to TMS and residual MeOD signals, respectively. Orbitrap Elite mass spectrometer equipped with HESI electrospray ion source (capillary temperature 275 °C, source heater temperature 40 °C; FTMS spray voltage 4.0 kV; resolution 60.000; Thermo Fisher Scientific, Munich, Germany) was used for high resolution ESI mass spectra measurements. Bruker Ultraflex III-MALDI-TOF/TOF mass spectrometer (Bruker Daltonics, Brussels, Belgium) was employed for MALDI-TOF mass spectra. The samples (1 µL) were mixed with the equal volume of 4 mg/mL α-cyano-4-hydroxycinnamic acid solution in 50% *v*/*v* acetonitrile/0.1% *v*/*v* trifluoroacetic acid (matrix) on a stainless-steel target and dried under air. The analysis was performed in a reflector positive ion mode, using the source and reflector voltages of 25 and 26.3 kV, respectively. Desorption and ionization of the analytes was achieved by a YAG 354 nm laser.

### 2.3. Synthesis of Dendrimers

#### 2.3.1. General Procedure for the Synthesis of Dendrimers **1a** and **2a**

PAMAM-NH_2_ dendrimer (81.2 µmol, 1.0 equivalent) was added to a round-bottom flask followed by isobutyraldehyde (0.32 mmol, 4.0 equivalent) in dry methanol. The reaction mixture was stirred at room temperature for 12 h to enable imine formation. CLB (0.32 mmol, 4.0 equivalent) and the isocyanide (0.32 mmol, 4.0 equivalent) were added and the contents were stirred for five days. The volatiles were removed under reduced pressure in a rotary evaporator. Crude product was purified by flash column chromatography (eluent: ethyl acetate/methanol).

#### 2.3.2. Synthesis of Dendrimer **1a**

PAMAM-NH_2_ dendrimer (43 mg, 81.2 µmol), isobutyraldehyde (22 mg, 0.32 mmol), CLB (98 mg, 0.32 mmol), and *tert*-butyl (2-(2-(2-isocyanoethoxy)ethoxy)ethyl)carbamate (46 mg, 0.32 mmol) were reacted in dry methanol (20 mL) according to section 2.3.1. The product was purified by flash column chromatography (SiO_2_, gradient elution, ethyl acetate/methanol 100:0 to ethyl acetate/methanol 50:50).

#### 2.3.3. Synthesis of Dendrimer **2a**

PAMAM-NH_2_ dendrimer (80 mg, 0.16 mmol), isobutyraldehyde (47 mg, 0.65 mmol), CLB (197 mg, 0.65 mmol), and methyl 4-isocyanobutanoate (83 mg, 0.65 mmol) were reacted in dry methanol (30 mL) according to section 2.3.1. The product was purified by flash column chromatography (SiO_2_, gradient elution, ethyl acetate/methanol 100:0 to ethyl acetate/methanol 50:50).

#### 2.3.4. Synthesis of Dendrimer **1b**

Dendrimer **1a** (30 mg, 0.01 mmol) was added to a round-bottom flask containing 750 µL of a CH_2_Cl_2_/TFA (4:1) solution (75 mL/mmol). The content was stirred for 2 h at room temperature, afterwards evaporated to dryness, and washed a few times with diethyl ether.

#### 2.3.5. Procedure for the Synthesis of Dendrimer **2b**

Dendrimer **2a** (100 mg, 0.04 mmol) was added to a round-bottomed flask containing a solution of THF/water 1:1 (5 mL/mmol) and NaOH (40 mg; 1 g/mmol). The contents were stirred for 18 h at room temperature and afterwards acidified with aqueous 5% HCl solution. The obtained salt was separated by filtration and the solution was concentrated under reduced pressure in a rotary evaporator.

#### 2.3.6. General Procedure for Synthesis of Dendrimers **3a**–**5a**

PAMAM dendrimer generation 0 (0.32 mmol, 1.0 equivalent) and the aldehyde (1.29 mmol, 4.0 equivalent) were added in a round-bottom flask in dry methanol. The mixture was stirred at room temperature overnight in order to accomplish imine formation. Then both biotin (0.65 mmol, 2.0 equivalent) and chlorambucil (0.65 mmol, 2.0 equivalent) were added followed by the isocyanide (1.29 mmol, 4.0 equivalent). The contents were stirred for 5 days at room temperature and the volatiles were removed under reduced pressure in a rotary evaporator. The products formed were pre-purified by flash column chromatography (SiO_2_, ethyl acetate/methanol 100:0 to ethyl acetate/methanol 0:100) followed by preparative RP-HPLC (AcN:H_2_O + 0.1% FA. 35% > 15 min > 80% > 1 min > 100%). Analytical data along with spectra are reported in the Supporting Information.

#### 2.3.7. General Procedure for the Synthesis of Dendrimers **3b**–**5b**

The dendrimer in THF/water 1:1 (*v*/*v*; 5 mL/mmol) and NaOH (1 g/mmol) were added in a round-bottom flask. The mixture was stirred overnight at room temperature and then acidified with aqueous HCl 5% solution. The salt formed was filtered off and the solution concentrated under reduced pressure in a rotary evaporator. For the synthesis of **3b**–**5b**, **3a**–**5a** dendrimers and NaOH were used as follows:

**3b**: **3a** (9.3 mg, 4 µmol) and NaOH (3 mg);
**4b**: **4a** (18.8 mg, 8 µmol) and NaOH (6.4 mg);
**5b**: **5a** (8 mg, 3 µmol) and NaOH (3 mg).

Analytical data along with spectra are reported in the Supporting Information.

### 2.4. Cell Lines and Culture Conditions

The cell lines selected for investigations were colon adenocarcinoma (HT-29), human refractory prostate cancer (PC-3), and mouse fibroblasts (NIH3T3). A complete medium, which consists of 10% FCS and 1% penicillin/streptomycin in RPMI 1640 medium, was used to grow the cells in an incubator at 37 °C and 5% CO_2_. The investigated dendrimers (**1a**/**b**–**5a**/**b**) were used to prepare a stock solution in DMSO of 20 mM concentration. Based on the surface area of the plates and the types of the cell lines used, the number of the seeded cells were selected. For 96-well plates, 1× 10^3^ PC-3, 1.5 × 10^3^ HT-29 cells, and 5 × 10^3^ NIH3T3 cells were seeded per well. While for the 6-well plates, 1 × 10^5^ PC-3 cells and 1.5 × 10^5^ HT-29 cells were seeded per well.

### 2.5. MTT and CV Assays

To identify anticancer active dendrimers, **1a**/**b**–**5a**/**b** were tested on HT-29 and PC-3 cell lines seeded in 96-well plates in two different concentrations of 0.01 and 10 µM. The treated cells were incubated for 72 h at 37 °C and 5% CO_2_. After incubation, the viability was determined using MTT and CV assays. After treatment, cells were exposed to 3-(4,5-dimethylthiazol-2-yl)-2,5-diphenyltetrazolium bromide solution (0.5 mg/mL) for 1 h, the MTT dye was removed, and the formazan formed was dissolved in DMSO. The absorbance was measured with a Spectramax plate reader (Molecular Devices, San Jose, CA, USA) at 570 nm with a reference wavelength of 670 nm. For the CV assay, after treatment cells were fixed with 4% paraformaldehyde for 10 min at room temperature and afterwards were stained for 15 min with 1% crystal violet solution. Cells were washed with water, dried, and the dye was dissolved in 33% acetic acid.

Dendrimer **1b,** which shows significant activity against the PC-3 cell line, as well as PAMAM-NH_2_, were further analyzed in a concentration series (1.65, 3.12, 6.25, 12.5, 25, 50, and 100 µM) to determine their IC_50_. The treated cells were incubated for 72 h at 37 °C and 5% CO_2_. The viability was checked using MTT and CV assays. Moreover, the activity of chlorambucil and **1b** against NIH3T3 was determined. Digitonin (125 µM) was used as a positive control. All experiments were performed in three technical and three biological replicates. The Spectramax plate reader (Molecular Devices, San Jose, CA, USA) was used for absorbance measurements (at 570 nm; reference at 670 nm) as described earlier [30]. For the calculation of the IC_50_ value, a four-parameter logistic function was used and the results presented as a mean of three independent trials.

### 2.6. Flow Cytometry

The most active dendrimer **1b** was selected for further analysis to determine its mechanism of action against prostate PC-3 cell line using a FACSAria III (DB Biosciences, Basel, Switzerland) flow cytometer.

#### 2.6.1. Cell Cycle Analysis

The effect of the most active compound **1b** on the cell cycle perturbation of the PC-3 cell line was determined by DAPI assay [12]. The cells were grown for 24 h in a 6-well plate, treated with the IC_50_ or 2 × IC_50_ concentration of **1b** for 72 h at 37 °C and 5% CO_2_. After incubation, the medium from the wells was collected and the cells were detached with 0.05% trypsin-EDTA. The detached cells were transferred to previously collected medium, washed with PBS, and fixed with 70% ethanol for 24 h. After fixation the cells were centrifuged, washed with PBS, and stained with 1 mL of DAPI working solution (1% Triton X-100, 1 µg/mL of DAPI in PBS) for 10 min. Afterwards, the samples were analyzed by flow cytometry.

#### 2.6.2. Apoptosis Assay

The extent of apoptosis induction by the most active compound was measured by AnnV/PI assay [12]. PC-3 cells were grown for 24 h in a 6-well plate and then treated with the IC_50_ or 2 × IC_50_ concentration of **1b**. Upon 72 h of incubation, the cells were collected by trypsination, and then stained with 100 µL AnnV/PI working solution (5% AnnV, 2% of PI in ABB), as indicated by the supplier. The cells were incubated at the room temperature for 15 min, and afterwards the stain was deactivated by the addition of 900 µL of ABB. The prepared samples were analyzed by flow cytometry.

#### 2.6.3. Caspase Activity Analysis

To determine if the caspases are involved in apoptosis, apostat assay was performed. PC-3 cells were grown for 24 h in a 6-well plate, treated with IC_50_ and 2 × IC_50_ concentration of **1b**, and incubated for 72 h at 37 °C and 5% CO_2_. The medium was discharged, cells were detached with 0.05% trypsin-EDTA, and collected. The collected cells were stained with 100 µL of apostat working solution (5% FCS, 1% Apostat in PBS) for 30 min at 37 °C and 5% CO_2_. The staining process was deactivated by the addition of 900 µL of PBS and the samples were analyzed by flow cytometry.

#### 2.6.4. Autophagy Analysis

PC-3 cells were plated in a 6-well plate and 24 h later treated for 72 h with IC_50_ and 2 × IC_50_ concentration of **1b**. Afterwards, the cells were stained with 500 µL of AO working solution (497 µL of PBS, 3 µL of 1 mM AO) for 15 min at 37 °C and 5% CO_2_. The staining was stopped by 1 mL of PBS and the samples were analyzed by flow cytometry.

#### 2.6.5. Cell Division Analysis

The impact of the **1b** dendrimer on PC-3 cell line was measured by CFSE assay [12]. Cells were prestained with CFSE working solution (1 µM CFSE in 0.1% FCS PBS). Afterwards, the cells were plated in a 6-well plate for 24 h. Then, the cells were treated with IC_50_ and 2 × IC_50_ concentration of **1b** (72 h). The cells were detached and resuspended in PBS for the analysis by flow cytometry.

#### 2.6.6. Investigation of NO Production

The NO production was analyzed with DAF-FM stain. Shortly, PC-3 cells were grown in a 6-well plate for 24 h and then treated with IC_50_ and 2 × IC_50_ concentrations of **1b** for 72 h. Afterwards, the cells were stained with 1 mL of DAF-FM working solution (5 µM DAF-FM diacetate in 10% FCS RPMI 1640) for 1 h at 37 °C and 5% CO_2_. Then, the stain was deactivated by incubation of the sample with a medium for 15 min. The cells were detached and then analyzed by flow cytometry.

#### 2.6.7. Statistical Analysis

Differences among results were evaluated by Student’s *t*-tests and were considered statistically significant for *p* values lower than 0.05.

## 3. Results and Discussion

### 3.1. Surface Functionalization of PAMAM-NH_2_ Dendrimer by Ugi Reaction

The surface modification of commercially available dendrimers via Ugi four-component reaction is based on the intrinsic characteristic of multicomponent reactions that allows a diversity-rich functionalization of macromolecules in a one-step procedure [31,32]. With this methodology, it is possible to add a new generation on the dendrimer surface with three distinct functionalities without previous modification or protection/deprotection strategies of the starting materials, which is normally required for existing procedures. [24,25].

**Scheme 1:**
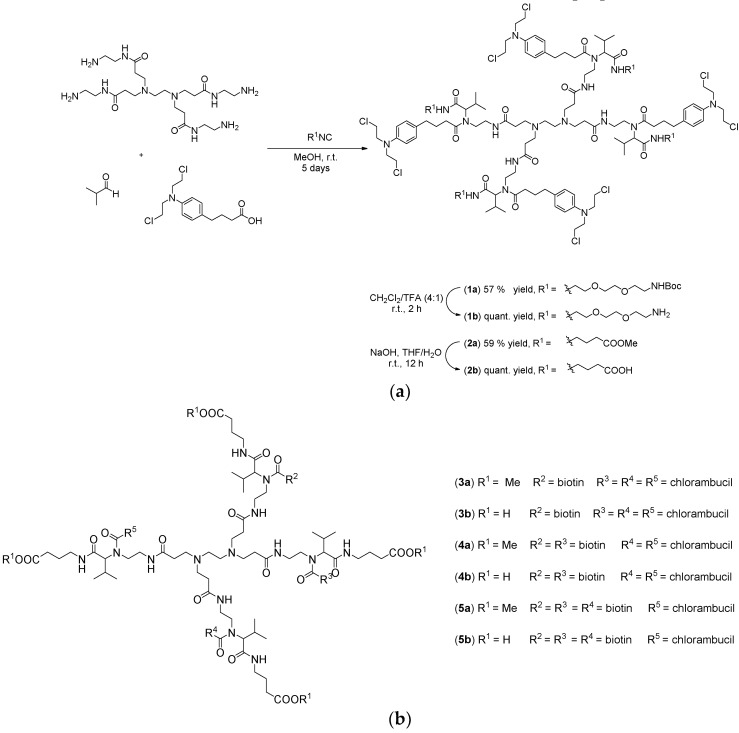
(**a**) Ugi reaction for the surface modification of PAMAM-NH_2_ dendrimer and (**b**) biotinylated chlorambucil-dendrimer conjugates (**3a**/**b**–**5a**/**b**) [33].

In this sense, PAMAM-NH_2_ dendrimer of generation 0 was selected as the amino component of the Ugi four-component reaction and as a water soluble nanocarrier for chlorambucil. As shown in Scheme 1a, two distinct Ugi reactions of PAMAM-NH_2_ dendrimer, isobutyraldehyde, and chlorambucil with two different isocyanides were performed, aiming to improve the water-solubility of the anticancer drug, while also adding cationic or anionic properties to the macromolecule. The one pot syntheses of the dendrimers were carried out by sequential addition of the building blocks. First, the amine and aldehyde were mixed together in dry methanol to enable formation of the imine. After 12 h, the acid component was added followed by the addition of the isocyanide. After the completion of the reaction, the products were purified by flash column chromatography using ethyl acetate/methanol as the eluent. Moreover, biotinylated chlorambucil-dendrimer conjugates, with the biotin and chlorambucil ratio 1:3 (**3a**/**b**), 2:2 (**4a**/**b**), 3:1 (**5a**/**b**), were also prepared (Scheme 1b).

The removal of the *tert*-butyloxycarbonyl and methyl protecting groups was accomplished with dichloromethane/trifluoroacetic acid (4:1) and sodium hydroxide, respectively, affording the desired water-soluble dendrimers in 57% (**1b**) and 59% (**2b**) overall yields.

The synthesized compounds were characterized by ^1^H and ^13^C NMR spectroscopy and mass spectrometry. All carbon and hydrogen atoms were undoubtedly assigned in the NMR spectra and the integrations are in accordance with the expected dendrimer structures. The ^1^H NMR spectra of compounds **1a**, **1b**, **2a**, and **2b** show two chemical shifts in the aromatic region (7.25–6.50 ppm) corresponding to 16 hydrogen atoms, thus confirming the presence of 4 chlorambucil units on the dendrimer surface. Besides that, it is possible to observe the characteristic resonances of PAMAM-NH_2_ core, isobutyraldehyde, and the isocyanides used in the Ugi reactions. Additionally, the removal of the protecting groups in **1b** and **2b** became apparent by the disappearance of the chemical shifts at 1.42 and 3.70 ppm, respectively (Figures S1, S2, S4, S5, Supporting information).

Moreover, the double charge or triple charge mass peaks of the dendrimers could be observed in the HRMS or MALDI-TOF spectra, confirming the identity of the products (Figures S3 and S5, Supporting information).

All dendrimers, after removal of the protecting groups, were obtained as water-soluble viscous colorless oils (Figure S31, Supporting information). Thus, the solubility of chlorambucil itself was improved, which is an important factor in order to increase the bioavailability and therapeutic efficacy of anticancer drugs.

### 3.2. Evaluation of Anticancer Activity

**Figure 1:**
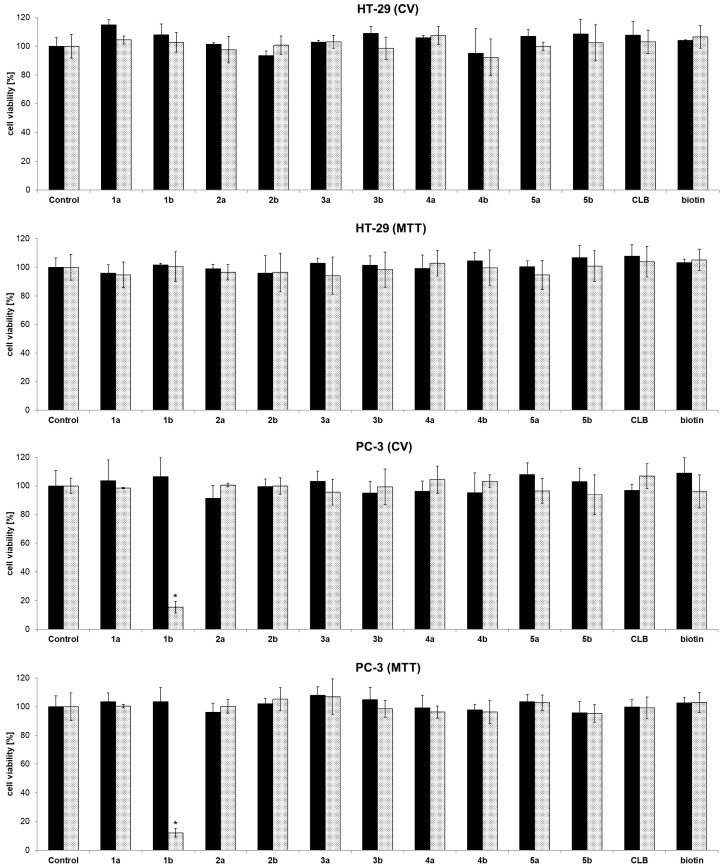
Fast screening of cell viability of control (vehicle without dendrimer), dendrimers **1a**/**b**–**5a**/**b**, chlorambucil (CLB), and biotin determined by CV and MTT assays against HT-29 and PC-3 cancer cell lines (72 h of action; █ 0.01 µM; ░ 10 µM) [33], * *p* < 0.05 refers to untreated cultures.

To assess the anticancer activity, the functionalized dendrimers were in vitro investigated against colon (HT-29) and prostate (PC-3) cancer cell lines (72 h). Controls were vehicle solution without dendrimer, and dendrimer without chlorambucil (“warhead”) as payload. In concordance with previous investigations, PAMAM-NH_2_ showed no activity (Figure S32, Supporting information) [34]. Fast screening of dendrimers at two basic concentrations (0.01 and 10 µM), as well as chlorambucil alone, demonstrated that the anticancer activity is dependent on the type of functional group present on the dendrimer surface (Figure 1). Namely, neutral (**1a**: –NHBoc; **2a**: –COOMe), and negatively charged dendrimers (**2b**: –COOH) were found inactive. Contrarily, the positively charged dendrimer (**1b**: –NH_2_) preferentially reduced PC-3 cell growth (>50%), whereas HT-29 cells were not affected at all (at a concentration of 10 µM).

The neutral or negatively charged dendrimers bearing chlorambucil, as well as PAMAM-NH_2_ dendrimer itself, exhibited no activity against the cancer cell lines investigated, which agrees with previous literature findings [35,36]. Biotinylated chlorambucil-dendrimer conjugates (**3a**/**b**–**5a**/**b**) were found inactive against tested cells. Results from different viability assays (CV and MTT) are in accordance with each other.

**Figure 2:**
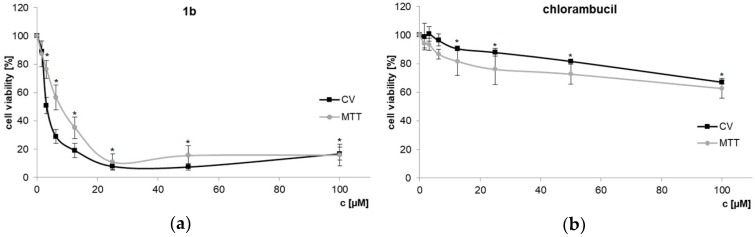
Dose-dependent response of PC-3 cells treated with dendrimer **1b** (**a**) and CLB (**b**). CV and MTT assays (72 h; 20 µM of dendrimer **1b** corresponds to 80 µM of chlorambucil in toxic moieties) [33], * *p* < 0.05 refers to untreated cultures.

In order to determine IC_50_ concentrations of dendrimer **1b** and chlorambucil, dose-dependent response against PC-3 prostate cancer cell line was explored using CV and MTT assays (Figure 2a). As previously demonstrated, chlorambucil itself shows only a low activity on PC-3 cells [32]. The cationic dendrimer **1b**, however, shows a considerable cytotoxic effect (IC_50_ values, CV: 3.65 ± 0.56 µM; MTT: 7.29 ± 1.18 µM), contrary to chlorambucil itself (CV, MTT: IC_50_ > 100 µM, Figure 2b). Even normalizing the data to the count of chlorambucil/molecule would mean that approximately 5 µM IC_50_ of dendrimer **1b** correlates to the four-fold concentration of unconjugated agent (i.e., 20 µM free chlorambucil), a concentration at which free chlorambucil still shows almost no activity.

**Figure 3:**
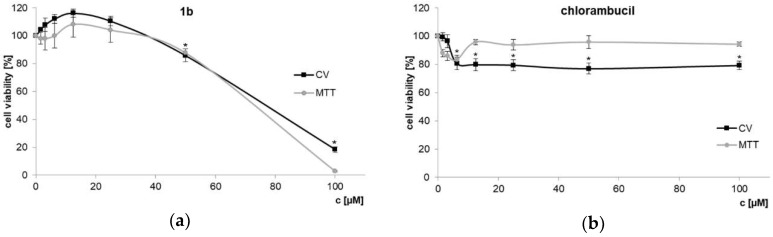
Non-cancerous mouse NIH3T3 fibroblasts treated with dendrimer **1b** (**a**) and chlorambucil (**b**) assessed by CV and MTT assays (72 h) * *p* < 0.05 refers to untreated cultures.

Additionally, NIH3T3 mouse fibroblasts were used to test toxic effects of dendrimer **1b** and chlorambucil on non-cancerous cells (Figure 3). Using **1b** at the IC_50_ determined for the PC-3 cell line (7.3 µM) did not affect NIH3T3 cell growth at all. The activity index between PC-3 and the non-cancerous cell line is > 10–20 (IC_50_ values NIH3T3, CV: 70.21 ± 1.11 µM; MTT: 74.37 ± 2.31 µM). HT-29 colon cancer cells, usually quite sensitive to cytotoxins, are likewise little effected (see Figure 1). This result clearly indicates that dendrimer **1b**, bearing four chlorambucil and four amino moieties, on one side boosted cytotoxicity and on the other improved discrimination toward the PC-3 prostate tumor cell line.

**Figure 4:**
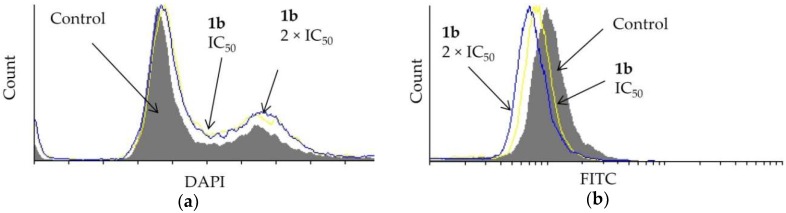
Effect of dendrimer **1b** at IC_50_ and 2 × IC_50_ concentrations (72 h) on PC-3 cells on (**a**) cell cycle distribution and (**b**) cell division.

Dendrimer **1b** affected the cell cycle distribution of prostate PC-3 cells (Figure 4a). A DAPI assay showed that the IC_50_ and the 2 × IC_50_ concentration of **1b** causes some entrapment of the cells in the G2/M-phase. Furthermore, the effect of **1b** on cell division was studied using a CFSE assay. The investigated dendrimer did not hinder the division of PC-3 cells (Figure 4b).

**Figure 5:**
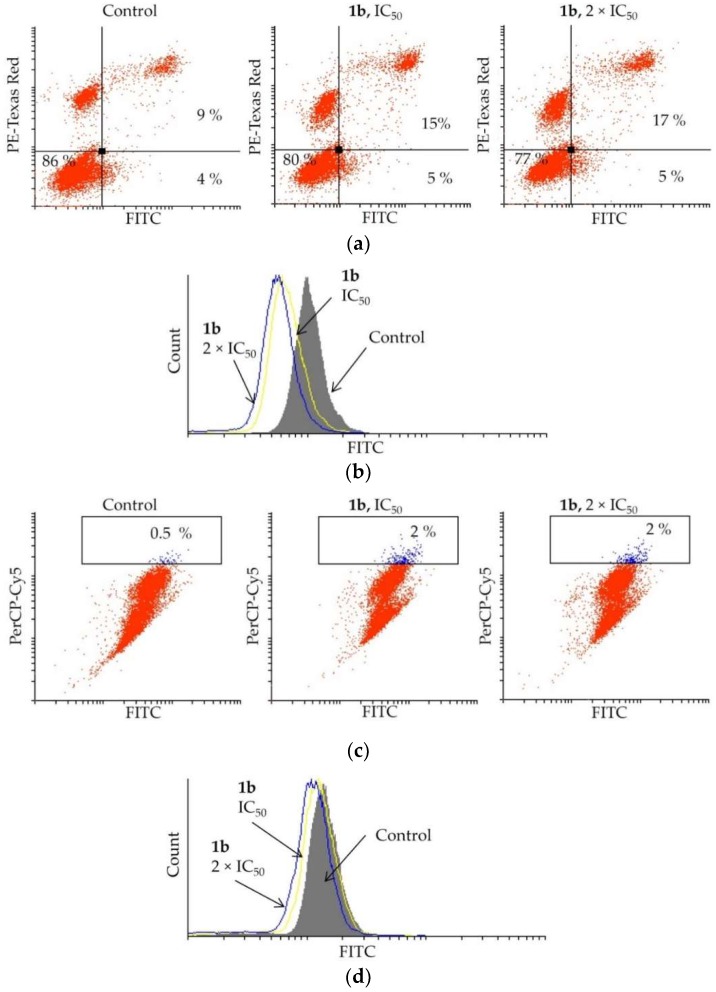
Effect of dendrimer **1b** upon treatment of PC-3 cells with IC_50_ and 2 × IC_50_ concentrations (72 h) on (**a**) induction of apoptosis, (**b**) caspase production, (**c)** induction of autophagy, and (**d**) NO production.

Elevated apoptosis was detected in PC-3 cells treated with **1b** (Figure 5a). By treating the cells with **1b** at IC_50_, the apoptotic events increased from 13 to 20%, and by doubling the concentration (2 × IC_50_), the number of apoptotic cells marginally increased further (22%). The mechanism of activation of apoptosis, either caspase dependent or independent, was determined by the apostat assay (Figure 5b). The flow cytometry analysis showed that compound **1b** suppresses caspase production in PC-3 cells, indicating that caspases are not involved in activating the apoptosis process.

As autophagy may mediate cell death, an AO assay was carried out upon treatment of PC-3 cells with dendrimer **1b**. The data obtained from the flow cytometry are presented in Figure 5c. Only an insignificant increase of the autophagosomes was observed when treating the PC-3 cells with compound **1b** at IC_50_ (control: 0.5%, **1b**: up to 2%). An increased concentration of **1b** (2 × IC_50_) also did not enhance formation of these acidic vesicles. To confirm that **1b** is not triggering autophagy in PC-3 cells, the effect of **1b** on NO production, a hallmark of autophagy occurrence, was investigated. The DAF-FM assay (Figure 5d) clearly showed only a slight reduction in the NO production. This undoubtedly indicates that autophagy is not activated considerably by the investigated dendrimer.

A plausible explanation for the superior behavior of dendrimer **1b** relies on the fact that polyamines (polycations) can interact via electrostatic attraction with the negatively charged phospholipids present on the membranes of living cells. This interaction permits the penetration of small and even quite large compounds into cells. However, in severe cases, such polyamines can also cause damage to, or rupture of, the cell membranes [37,38]. Although this does not fully explain the selectivity for PC-3 cancer cell lines, some authors pointed out that certain amines are preferentially absorbed by some types of prostate cells [22,23]. Here we can only assume that PC-3 cells recognize the cationic dendrimer surface of **1b** as suitable for selective uptake, while the amino core dendrimers (see other derivatives) are obviously not recognized. In other contexts, dendrimers have been already proven beneficial for selective uptake [39]. However, further studies are necessary to better understand this behavior. Nevertheless, in our study, the polyamine dendrimer **1b** appears to favor uptake into the prostate PC-3 cancer cell line, while NIH3T3 fibroblasts or HT-29 colon cells appear to be insensitive or less susceptible to interaction with polycationic chlorambucil-dendrimer conjugate.

In order to improve the cellular uptake of the chlorambucil-dendrimer conjugates, three water soluble dendrimers containing different ratios of the anticancer drug chlorambucil and the targeting molecule biotin in the same molecule were also synthesized by Ugi reaction (Scheme 1b and Table 1). The one pot procedure was performed employing PAMAM-NH_2_ dendrimer, isobutyraldehyde, both biotin and chlorambucil, and methyl 4-isocyanobutanoate. Surprisingly, the fast screening against colon HT-29 and prostate PC-3 cancer cell lines demonstrated that those dendrimers are inactive in the concentration range tested (Figure 1).

## 4. Conclusions

For the first time, a MCR-strategy was successfully used to functionalize a PAMAM-NH_2_ dendrimer and to enhance the activity and solubility of a chemotherapeutic drug. We showed that the new dendrimer generation introduced by the Ugi-branching method allows the simultaneous and multiple introduction of –COOH, –NH_2_, biotin, or lipidic surface groups, in addition to the cytotoxic payload in a one pot process. The evaluation of the cytotoxic activity against HT-29 colon and PC-3 prostate cancer cell lines showed that the polycationic dendrimer with four CLB units on the surface (**1b**) preferentially improves the anticancer activity against the hard to treat PC-3 prostate cancer cell line, blocking the G2/M phase and inducing caspase independent apoptosis. Dendrimer **1b** was found to be selective not only against another tumor cell type, but more importantly, also against a non-cancerous cell line (mouse fibroblasts). Moreover, the cytotoxicity of **1b** is not solely due to the presence of four chlorambucil moieties on the dendrimer surface, as chlorambucil alone was found inactive even at 4-fold concentration, as was the dendrimer core alone. Only the proper combination of dendrimer core, payload, and additional cationic (amino) surface groups produces a sufficiently active candidate compound.

## Figures and Tables

**Table 1:** List of all synthesized dendrimers, including the ratio of chlorambucil (*n*) and biotin (*m*) per dendrimer molecule, solubility (at 20 mg/mL), and overview of anticancer activity (at ≤ 10 µM) against HT-29 colon and PC-3 prostate cancer cell lines.

Dendrimer	CLB *n*	Biotin *m*	Solubility Tag Present in the Dendrimers Structure	Solubility	HT-29	PC-3
**1a**	4	0	NHBoc	Low	Inactive	Inactive
**1b**	4	0	NH_2_	High	Inactive	Active
**2a**	4	0	COOMe	Low	Inactive	Inactive
**2b**	4	0	COOH	High	Inactive	Inactive
**3a**	3	1	COOMe	Low	Inactive	Inactive
**3b**	3	1	COOH	High	Inactive	Inactive
**4a**	2	2	COOMe	Low	Inactive	Inactive
**4b**	2	2	COOH	High	Inactive	Inactive
**5a**	1	3	COOMe	Low	Inactive	Inactive
**5b**	1	3	COOH	High	Inactive	Inactive

## Supplementary Materials

The following information are available online at http://www.mdpi.com/1999-4923/11/2/59/s1, Figure S1. ^1^H NMR spectrum of compound **1a**. Figure S2. ^13^C NMR spectrum of compound **1a**. Figure S3. HRMS spectrum obtained for compound **1a**. Figure S4. ^1^H NMR spectrum of compound **1b**. Figure S5. ^13^C NMR spectrum of compound **1b**. Figure S6. MALDI-TOF spectrum of compound **1b**. Figure S7. ^1^H NMR spectrum of compound **2a**. Figure S8. ^13^C NMR spectrum of compound **2a**. Figure S9. HRMS spectrum of compound **2a**. Figure S10. ^1^H NMR spectrum of compound **2b**. Figure S11. ^13^C NMR spectrum of compound **2b**. Figure S12. MALDI-TOF spectrum of compound **2b**. Figure S13. ^1^H NMR spectrum of compound **3a**. Figure S14. ^13^C NMR spectrum of compound **3a**. Figure S15. MALDI-TOF spectrum of compound **3a**. Figure S16. ^1^H NMR spectrum of compound **3b**. Figure S17. ^13^C NMR spectrum of compound **3b**. Figure S18. MALDI-TOF spectrum (expansion of *m*/*z* 2337–2384) of compound **3b**. Figure S19. ^1^H NMR spectrum of compound **4a**. Figure S20. ^13^C NMR spectrum of compound **4a**. Figure S21. MALDI-TOF spectrum (expansion) of compound **4a**. Figure S22. ^1^H NMR spectrum of compound **4b**. Figure S23. ^13^C NMR spectrum of compound **4b**. Figure S24. MALDI-TOF spectrum obtained for compound **4b**. Figure S25. ^1^H NMR spectrum of compound **5a**. Figure S26. ^13^C NMR spectrum of compound **5a**. Figure S27. MALDI-TOF spectrum of compound **5a**. Figure S28. ^1^H NMR spectrum of compound **5b**. Figure S29. ^13^C NMR spectrum of compound **5b**. Figure S30. HRMS spectrum obtained for compound **5b**. Figure S31. Water solubility—compound **1b** before (a) and after dissolution in de-ionized water (b). Figure S32. Dose-dependent response of PC-3 cells treated with PAMAM-NH_2_, CV, and MTT assays (72 h).
