# Supplementary material for: Chlorambucil Conjugated Ugi Dendrimers with PAMAM-NH2 Core and Evaluation of Their Anticancer Activity
Source: Pharmaceutics. 2019 Feb 1;11(2):59. doi: 10.3390/pharmaceutics11020059 (PMC6409784; doi:10.3390/pharmaceutics11020059)
Supplement: Supplementary file 1 [file pharmaceutics-11-00059-s001.pdf]

pharmaceutics

# Supplementary Materials: Chlorambucil Conjugated Ugi Dendrimers with PAMAM-$NH_2$ Core and Evaluation of Their Anticancer Activity

**Nalin Seixas, Bruno B. Ravanello, Ibrahim Morgan, Goran N. Kaluđerović and Ludger A. Wessjohann**

## 1. Analytical Data and Spectra of Dendrimers

**1a**: Colorless oil. Yield: 138 mg (57 %). HRMS (ESI) of $C_{142}H_{236}Cl_8N_{22}O_{28}$ $[M+3H]^{3+}$ calc. 993.5156, obs. 993.8508; $^1H$ NMR (400 MHz, MeOD) δ 7.06 (d, 8H, *J* = 7.8 Hz), 6.66 (d, 8H, *J* = 12.8 Hz), 4.52 (d, 4H, *J* = 11.0 Hz), 3.76–3.32 (m, 88H), 3.22–3.17 (m, 8H), 2.91–2.78 (m, 8H), 2.62–2.16 (m, 32H), 1.94–1.85 (m, 8H), 1.42 (s, 36H), 0.95–0.79 (m, 24H); $^{13}C$ NMR (101 MHz, MeOD) δ 176.90, 172.68, 158.34, 145.99, 131.80, 130.67, 113.60, 80.06, 71.29, 71.08, 70.44, 54.55, 54.53, 48.85, 41.79, 41.27, 41.21, 40.38, 40.29, 40.03, 35.20, 35.06, 34.98, 33.66, 28.97, 28.84, 28.43, 28.28, 20.15, 19.88, 19.80, 19.41.

**1b**: Yield: 25 mg (quantitative). MS (MALDI-TOF) of $C_{122}H_{204}Cl_8N_{22}O_{20}$ $[M+Na]^+$ calc. 2600.302, obs. 2603.291; $^1H$ NMR (400 MHz, MeOD) δ 7.05 (d, 8H, *J* = 8.0 Hz), 6.66 (d, 8H, *J* = 7.2 Hz), 4.47 (d, 4H, *J* = 11.2 Hz), 3.73–3.32 (m, 88H), 3.24–3.09 (m, 20H), 2.61–2.48 (m, 16H), 2.27–2.19 (m, 4H), 1.92–1.83 (m, 8H), 1.34–1.28 (m, 8H), 0.96–0.79 (m, 24H); $^{13}C$ NMR (101 MHz, MeOD) δ 176.96, 176.26, 172.79, 172.15, 146.07, 131.73, 130.76, 130.67, 130.65, 130.56, 126.11, 113.75, 113.72, 113.63, 113.56, 71.34, 71.29, 71.24, 70.44, 67.91, 54.50, 54.32, 49.84, 41.79, 40.68, 40.57, 40.23, 40.18, 35.14, 35.10, 35.05, 34.96, 33.65, 30.89, 28.91, 28.44, 28.23, 20.07, 19.75, 19.67, 19.65, 19.42.

**2a**: Colorless oil. Yield: 235 mg (59 %). HRMS (ESI) of $C_{118}H_{184}Cl_8N_{18}O_{20}$ $[M+2H]^{2+}$ calc. 1227.5801, obs. 1228.5787; $^1H$ NMR (400 MHz, MeOD) δ 7.07–7.03 (m, 8H), 6.68–6.64 (m, 8H), 4.50 (d, 4H, *J* = 11.0 Hz), 3.74–3.32 (m, 64H), 3.28–3.13 (m, 12H), 2.97–2.88 (m, 4H), 2.57–2.21 (m, 36H), 1.90–1.75 (m, 16H), 0.97–0.75 (m, 24H); $^{13}C$ NMR (101 MHz, MeOD) δ 179.58, 176.89, 176.12, 175.09, 175.04, 172.60, 171.92, 146.01, 145.91, 132.13, 131.85, 131.79, 131.77, 130.68, 130.64, 130.58, 113.72, 113.61, 113.59, 113.55, 113.53, 113.37, 67.71, 54.61, 54.54, 54.52, 52.19, 52.15, 49.84, 41.78, 41.76, 41.70, 40.09, 39.75, 39.58, 35.90, 35.31, 35.15, 35.03, 33.61, 32.07, 32.02, 29.42, 28.87, 28.76, 28.38, 28.28, 27.85, 25.55, 20.09, 19.85, 19.66, 19.37.

**2b**: Yield: 96 mg (quantitative). MS (MALDI-TOF) of $C_{114}H_{176}Cl_8N_{18}O_{20}$ $[M+2H+Na]^{3+}$ calc. 807.026, obs. 807.704; $^1H$ NMR (400 MHz, MeOD) δ 7.10 (d, 8H, *J* = 8.6 Hz), 6.77 (d, 8H, *J* = 8.4 Hz), 4.58–4.46 (m, 4H), 3.78–3.60 (m, 52H), 3.29–3.17 (m, 13H), 2.90–2.85 (m, 4H), 2.63–2.24 (m, 36H), 1.90–1.77 (m, 17H), 0.96–0.78 (m, 24H); $^{13}C$ NMR (101 MHz, MeOD) δ 177.44, 176.76, 176.75, 176.70, 176.68, 176.61, 176.15, 176.03, 172.61, 172.56, 171.95, 171.90, 145.20, 145.20, 145.12, 131.60, 131.53, 130.78, 130.74, 130.69, 114.41, 114.39, 114.36, 114.34, 114.28, 114.24, 114.22, 114.18, 68.84, 67.50, 65.38, 55.07, 54.93, 49.84, 41.54, 41.37, 39.78, 39.68, 35.39, 35.05, 35.01, 34.22, 34.10, 33.81, 33.63, 32.17, 32.08, 32.00, 28.42, 28.03, 27.92, 26.48, 25.60, 20.11, 19.79, 19.46, 19.39, 19.21.

**3a**: Yield: 23.3 mg. MS (MALDI-TOF) of $C_{114}H_{181}Cl_6N_{19}O_{21}S$ $[M+Na]^+$ calc. 2417.142, obs. 2419.247; $^1$H NMR (400 MHz, MeOD) δ 7.09–7.03 (m, 6H), 6.67 (dd, 6H, *J* = 8.5, 4.2 Hz), 4.52–4.45 (m, 4H), 4.33–4.27 (m, 2H), 3.73–3.60 (m, 36H), 3.43–3.34 (m, 8H), 3.29–3.11 (m, 16H), 2.85–2.20 (m, 57H), 1.91–1.88 (m, 4H), 1.81–1.77 (m, 6H), 1.29–1.28 (m, 3H), 0.94–0.79 (m, 24H); $^{13}$C NMR (101 MHz, MeOD) δ 177.01, 176.98, 175.11, 175.07, 172.61, 172.56, 146.02, 131.81, 130.65, 113.62, 54.57, 52.52, 52.18, 52.14, 51.28, 51.24, 51.20, 41.79, 40.10, 40.02, 39.58, 39.53, 35.16, 34.52, 33.63, 33.61, 32.10, 32.03, 28.92, 28.35, 28.28, 25.56, 20.10, 19.88, 19.71, 19.36.

**3b**: Yield: 9 mg (quantitative). MS (MALDI-TOF) of $C_{110}H_{173}Cl_6N_{19}O_{21}S$ $[M+Na]^+$ calc. 2361.079, obs. 2363.169; $^1$H NMR (400 MHz, MeOD) δ 7.06 (d, 6H, *J* = 8.2 Hz), 6.70–6.64 (m, 6H), 4.56–4.47 (m, 4H), 4.34–4.26 (m, 2H), 3.72–3.62 (m, 24H), 3.44–3.34 (m, 8H), 3.24–3.08 (m, 16H), 2.60–1.74 (m, 63H), 1.32–1.29 (m, 6H), 0.95–0.79 (m, 24H); $^{13}$C NMR (101 MHz, MeOD) δ 176.72, 176.65, 176.22, 172.66, 146.06, 131.84, 131.75, 130.68, 130.63, 113.62, 54.59, 54.54, 41.80, 41.78, 39.87, 39.72, 35.12, 35.02, 33.66, 33.58, 32.24, 32.18, 30.75, 28.77, 28.41, 28.33, 25.63, 23.72, 20.85, 20.73, 20.06, 19.84, 19.61, 19.37.

**4a**: Yield: 26.3 mg. MS (MALDI-TOF) of $C_{110}H_{178}Cl_4N_{20}O_{22}S_2$ $[M+Na]^+$ calc. 2358.151, obs. 2360.288; $^1$H NMR (400 MHz, MeOD) δ 7.08–7.04 (m, 4H), 6.70–6.65 (m, 4H), 4.56–4.45 (m, 6H), 4.33–4.27 (m, 2H), 3.75–3.57 (m, 36H), 3.41–3.34 (m, 6H), 3.24–3.16 (m, 12H), 2.93–2.83 (m, 8H), 2.73–2.55 (m, 16H), 2.45–2.15 (m, 24H), 1.91–1.76 (m, 14H), 1.68–1.47 (m, 10H), 0.95–0.79 (m, 24H); $^{13}$C NMR (101 MHz, MeOD) δ 177.11, 177.06, 176.92, 175.10, 175.06, 172.60, 172.56, 166.01, 165.95, 146.03, 146.01, 131.86, 131.78, 130.69, 130.65, 113.73, 113.60, 54.55, 54.52, 52.20, 52.17, 52.16, 41.81, 41.79, 41.24, 41.10, 40.12, 40.03, 39.75, 39.57, 35.15, 35.02, 33.91, 33.76, 33.62, 32.11, 32.08, 32.03, 29.93, 29.77, 29.68, 29.61, 29.41, 28.92, 28.38, 28.29, 27.15, 27.04, 25.55, 20.10, 20.06, 19.87, 19.69, 19.37, 19.27.

**4b**: Yield: 18 mg (quantitative). MS (MALDI-TOF) of $C_{106}H_{170}Cl_4N_{20}O_{22}S_2$ $[M+K]^+$ calc. 2318.197, obs. 2318.053; $^1$H NMR (400 MHz, MeOD) δ 7.08–7.04 (m, 4H), 6.70–6.66 (m, 4H), 4.59–4.42 (m, 6H), 4.35–4.27 (m, 2H), 3.72–3.62 (m, 24H), 3.42–3.33 (m, 6H), 3.26–3.05 (m, 20H), 2.60–2.25 (m, 30H), 1.92–1.59 (m, 24H), 1.47–1.29 (m, 10H), 0.98–0.77 (m, 24H); $^{13}$C NMR (101 MHz, MeOD) δ 176.87, 146.06, 130.68, 130.63, 130.57, 130.11, 113.62, 113.56, 54.54, 51.26, 41.79, 41.69, 39.91, 39.71, 35.79, 35.20, 35.04, 34.24, 33.64, 32.32, 30.76, 29.74, 28.34, 28.05, 25.69, 20.05, 19.84, 19.52, 19.36.

**5a**: Yield: 16 mg. MS (MALDI-TOF) of $C_{106}H_{175}Cl_2N_{21}O_{23}S_3$ $[M+Na]^+$ calc. 2299.159, obs. 2300.261; $^1$H NMR (400 MHz, MeOD) δ 7.09–7.04 (m, 2H), 6.70–6.66 (m, 2H), 4.56–4.46 (m, 6H), 4.36–4.29 (m, 4H), 3.73–3.61 (m, 20H), 3.42–3.33 (m, 8H), 3.29–3.13 (m, 16H), 2.96–2.90 (m, 3H), 2.80–2.57 (m, 18H), 2.41–2.13 (m, 22H), 1.88–1.28 (m, 36H), 0.97–0.80 (m, 24H); $^{13}$C NMR (101 MHz, MeOD) δ 177.17, 177.13, 175.36, 175.13, 172.59, 172.57, 166.04, 165.99, 146.04, 131.82, 130.66, 113.62, 64.36, 63.48, 61.58, 57.12, 54.58, 52.17, 41.82, 41.22, 40.10, 40.02, 39.76, 39.58, 35.16, 34.65, 34.50, 33.96, 32.13, 32.04, 29.93, 29.80, 29.70, 29.63, 28.29, 27.12, 27.04, 25.56, 20.07, 19.88, 19.70, 19.36, 19.31, 19.27.

**5b**: Yield: 7 mg (quantitative). HRMS (ESI) of $C_{102}H_{167}Cl_2N_{21}O_{23}S_3$ $[M+Na+K]^{2+}$ calc. 1141.097, obs. 1141.092; $^1$H NMR (400 MHz, MeOD) δ 7.27–7.24 (m, 2H), 7.11–7.06 (m, 2H), 4.62–4.31 (m, 10H), 3.92–3.85 (m, 8H), 3.67–3.59 (m, 14H), 3.26–3.19 (m, 10H), 2.97–2.24 (m, 43H), 1.94–1.30 (m, 36H), 0.97–0.78 (m, 24H); $^{13}$C NMR (101 MHz, MeOD) δ 176.97, 176.62, 175.21, 172.60, 171.94, 165.86, 138.67, 131.20, 129.66, 128.85, 117.83, 67.52, 65.36, 63.67, 57.18, 56.84, 52.22, 49.84, 46.05, 41.03, 40.37, 39.71, 39.58, 35.27, 34.54, 32.03, 29.91, 29.67, 29.43, 28.27, 25.89, 25.54, 20.12, 19.77, 19.42, 19.34.

## 2. Analytical Data

Note: NMR spectra in the Ugi part might show mixtures of E/Z-amides and diastereomers where applicable, i.e., multiple or broadened peaks.

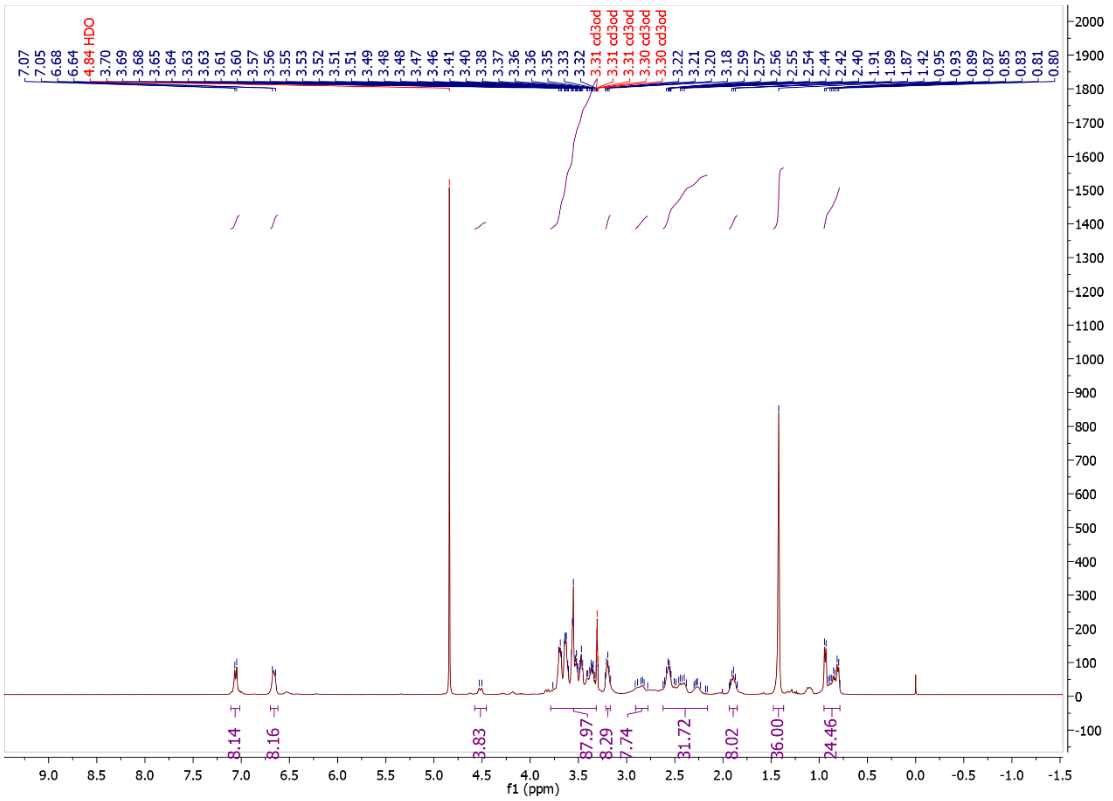

**Figure S1.** $^{1}H$ NMR spectrum of compound **1a**.

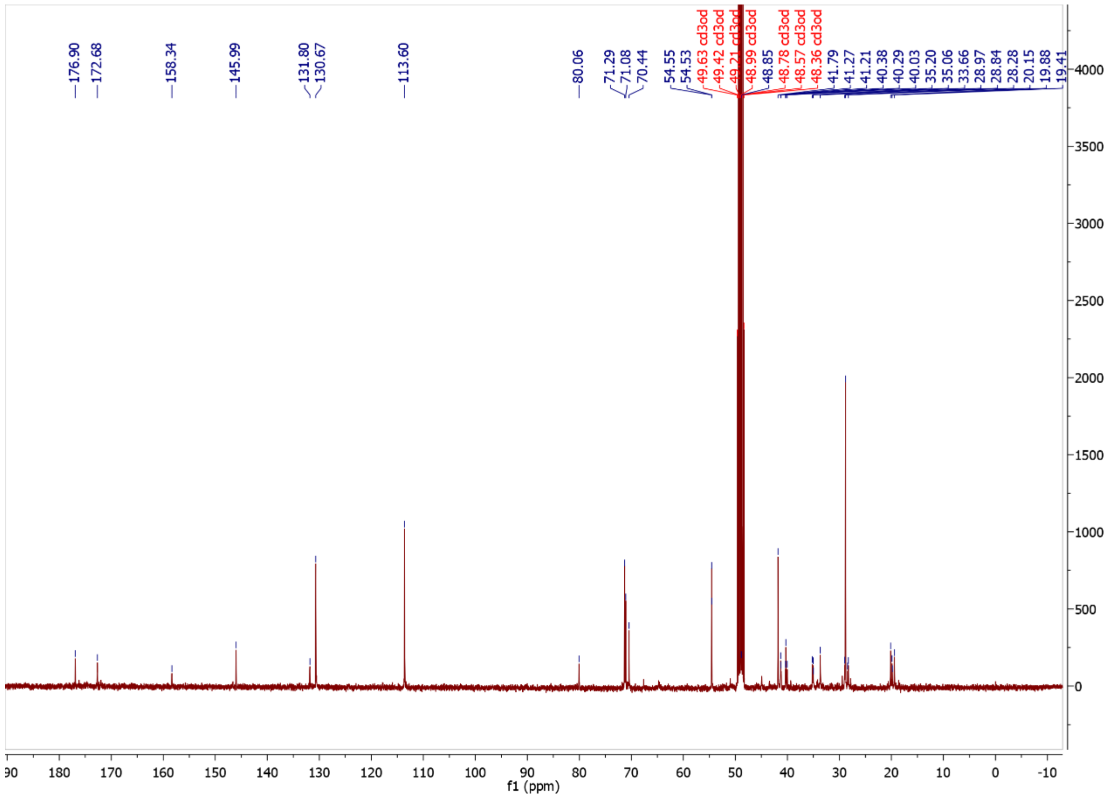

**Figure S2.** $^{13}C$ NMR spectrum of compound **1a**.

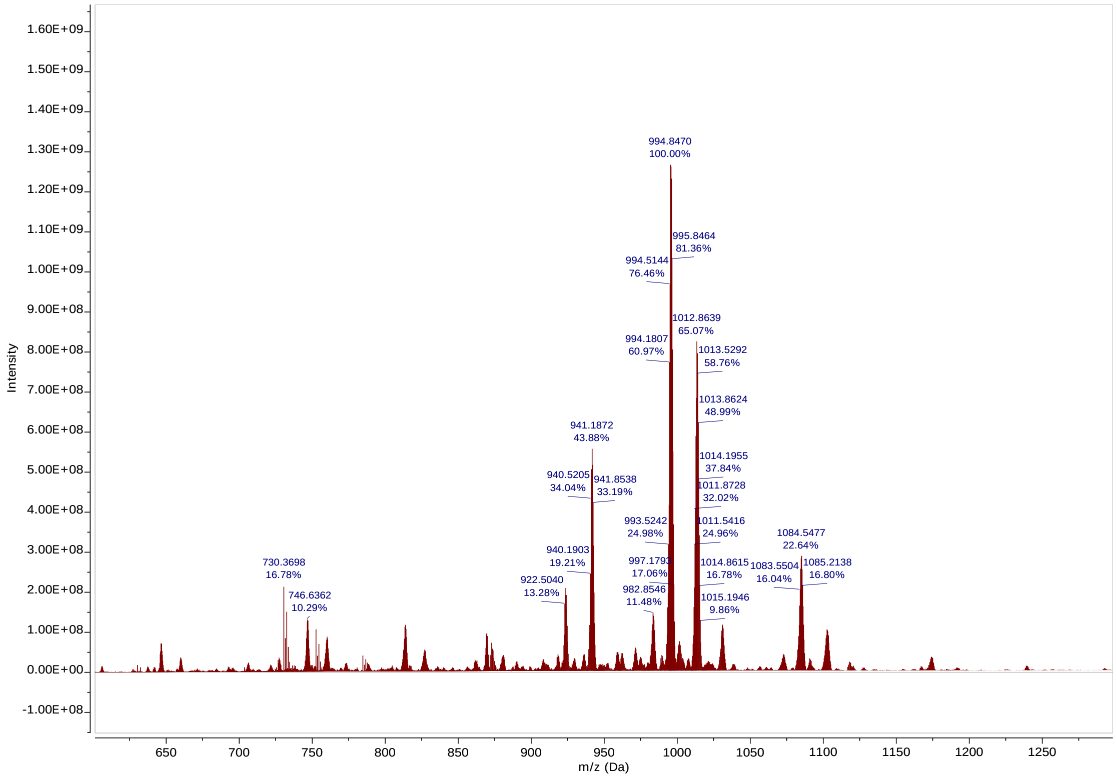


**Figure S3.** HRMS spectrum obtained for compound **1a**.

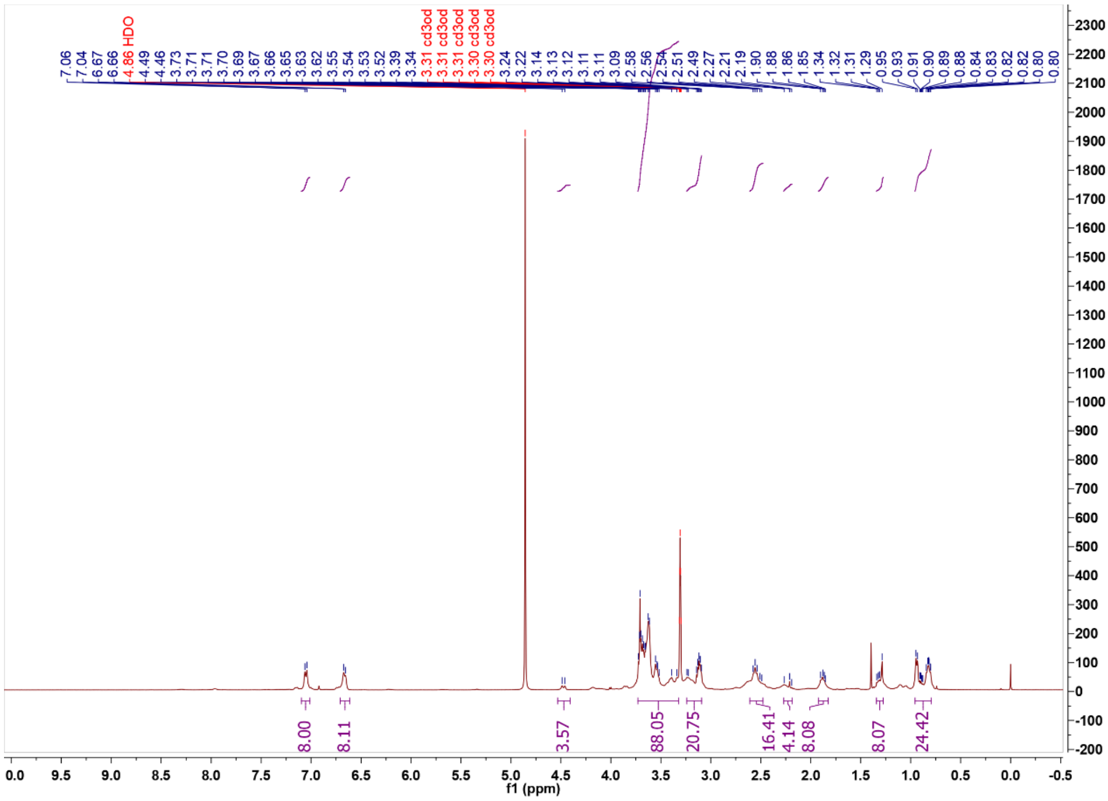


**Figure S4.** $^{1}$H NMR spectrum of compound **1b**.

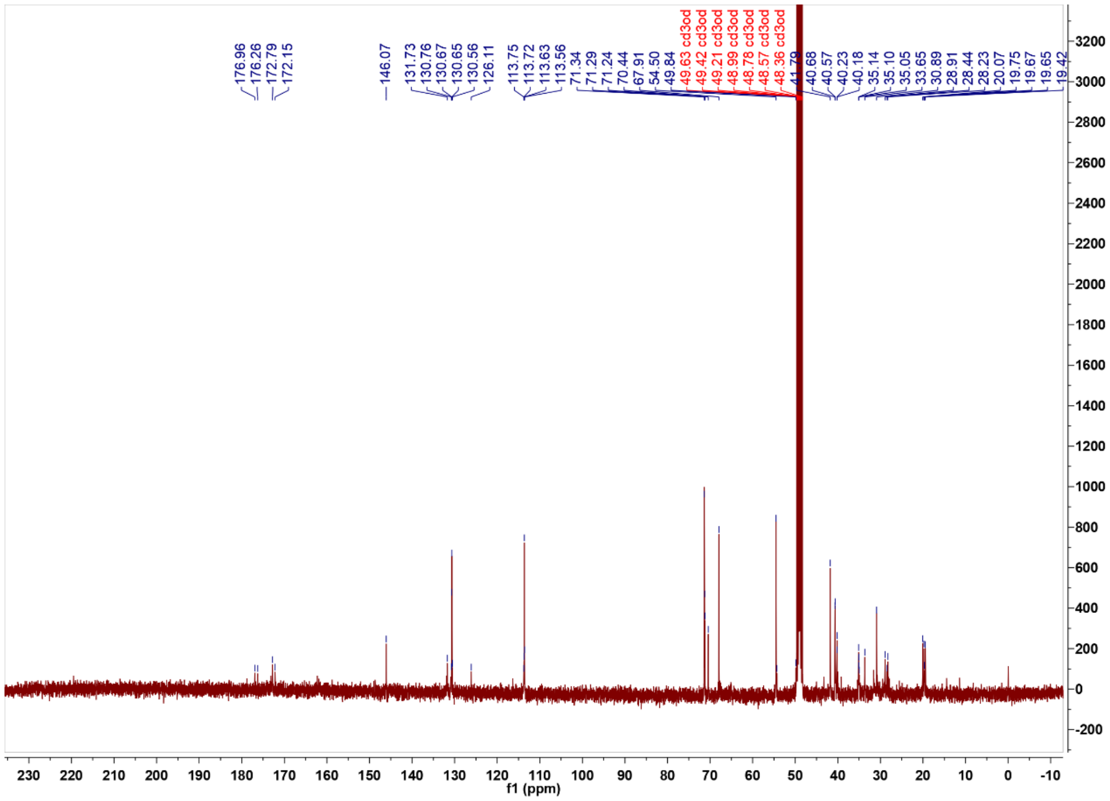


**Figure S5.** $^{13}C$ NMR spectrum of compound **1b**.

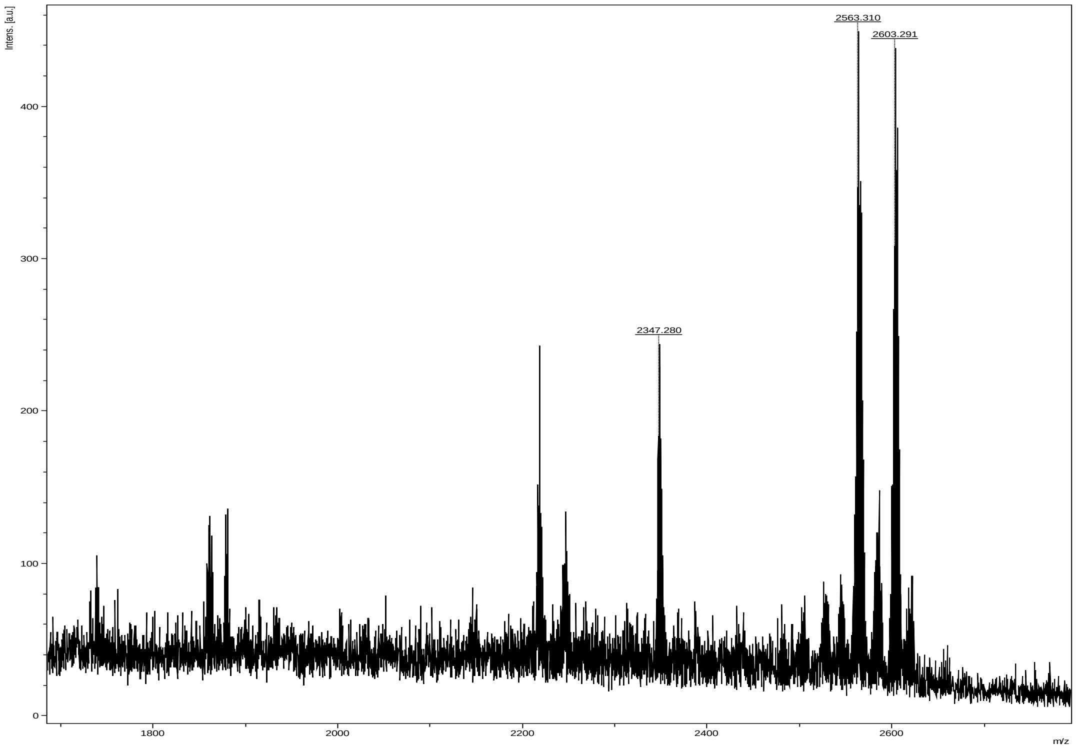


**Figure S6.** MALDI-TOF spectrum of compound **1b**.

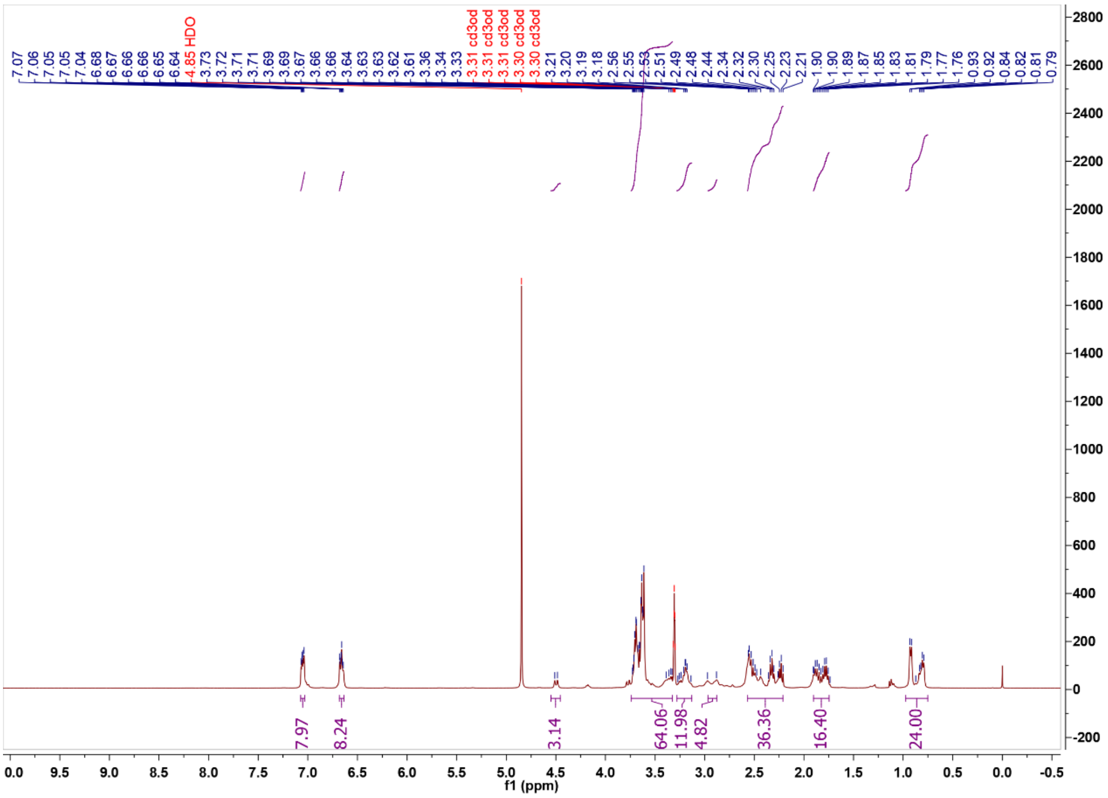

**Figure S7.** $^{1}H$ NMR spectrum of compound **2a**.

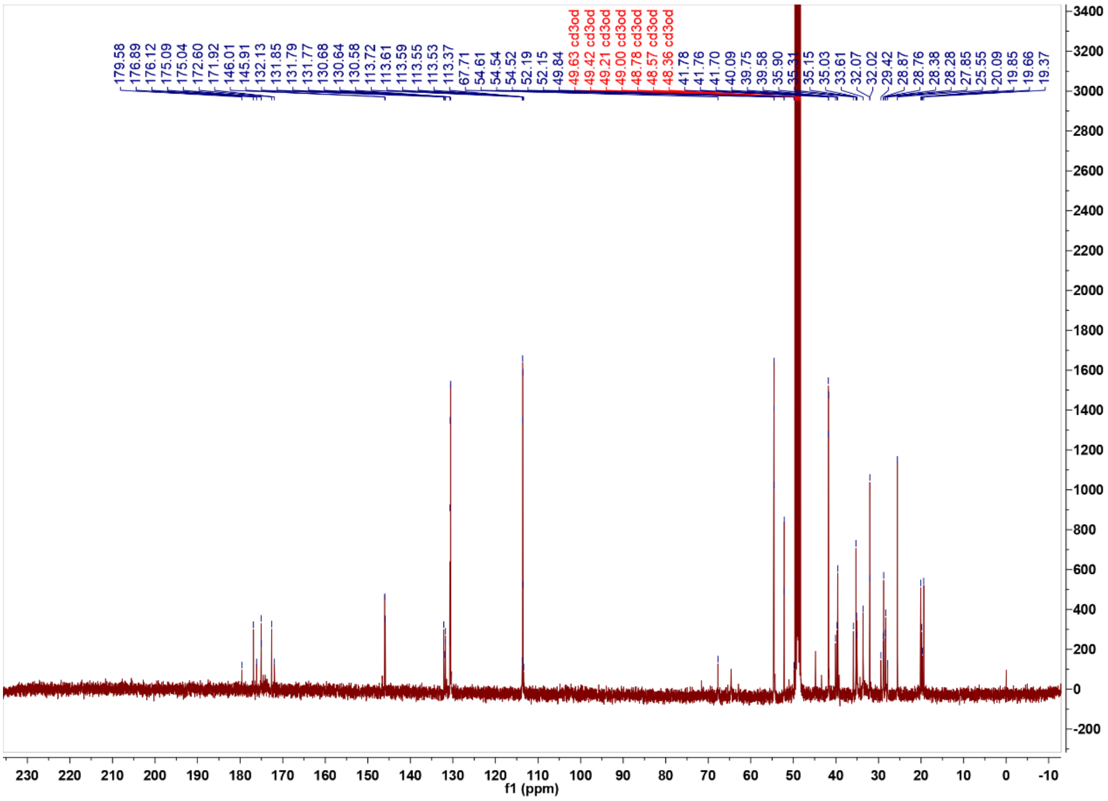

**Figure S8.** $^{13}C$ NMR spectrum of compound **2a**.

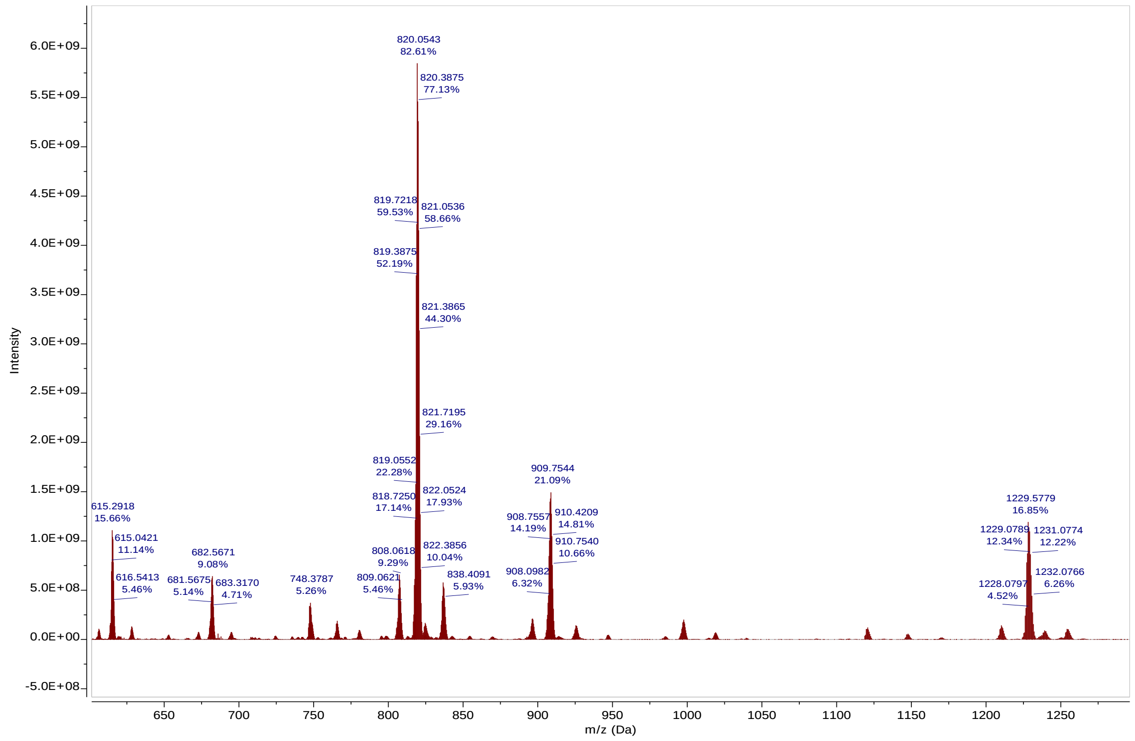

**Figure S9.** HRMS spectrum of compound **2a**.

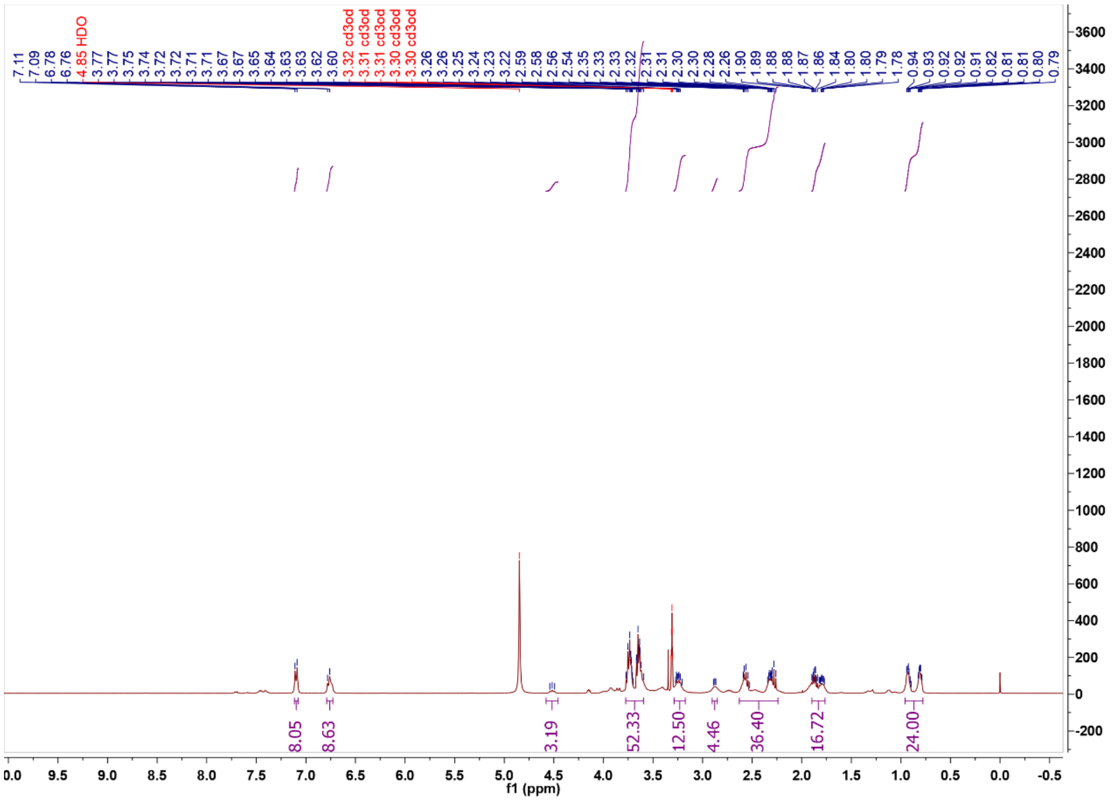

**Figure S10.** $^{1}$H NMR spectrum of compound **2b**.

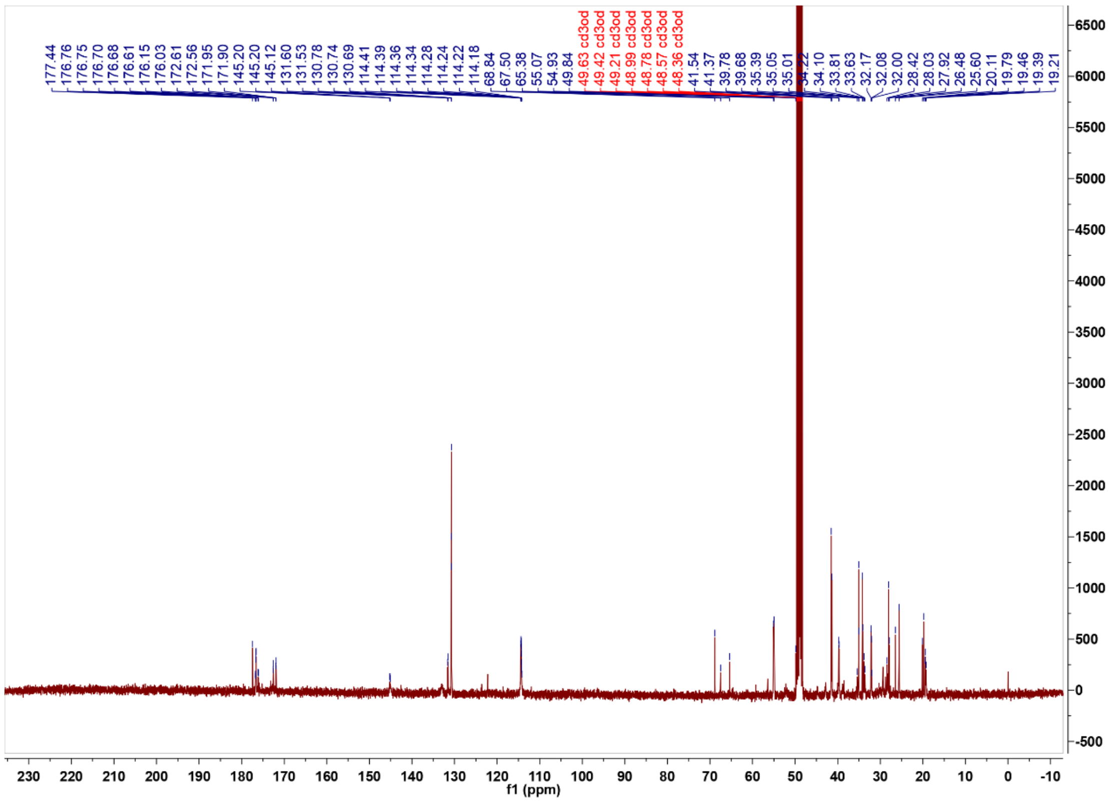


**Figure S11.** $^{13}C$ NMR spectrum of compound **2b**.

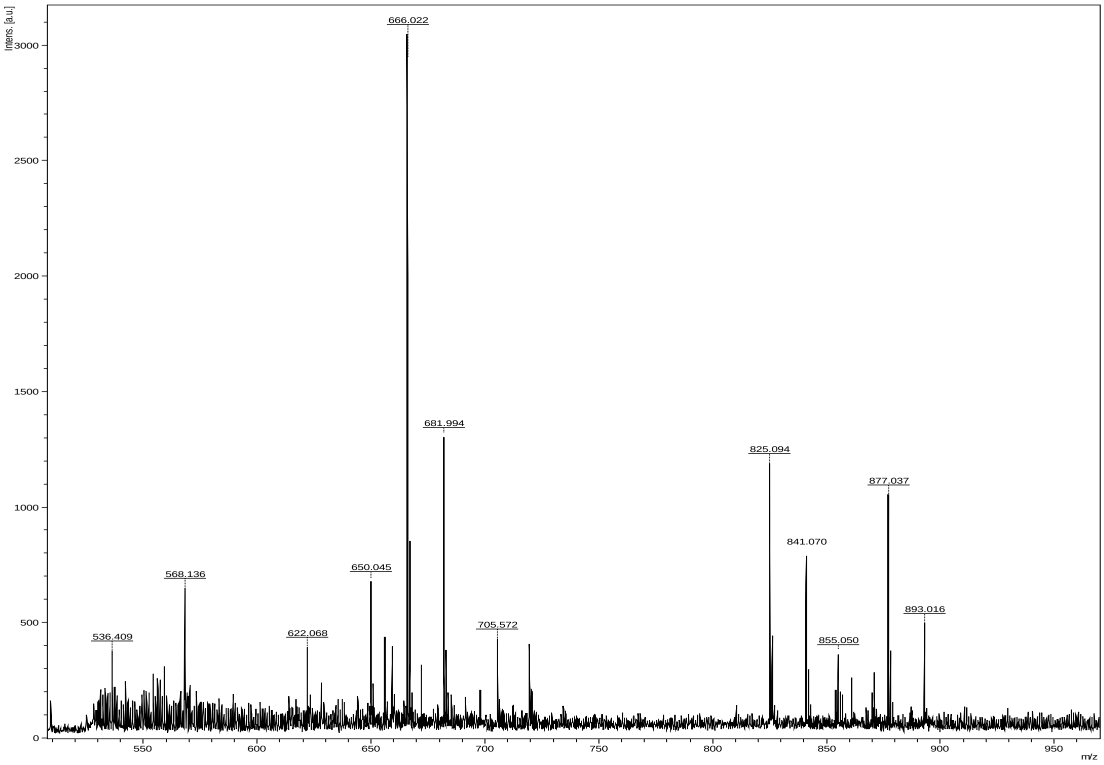


**Figure S12.** MALDI-TOF spectrum of compound **2b**.

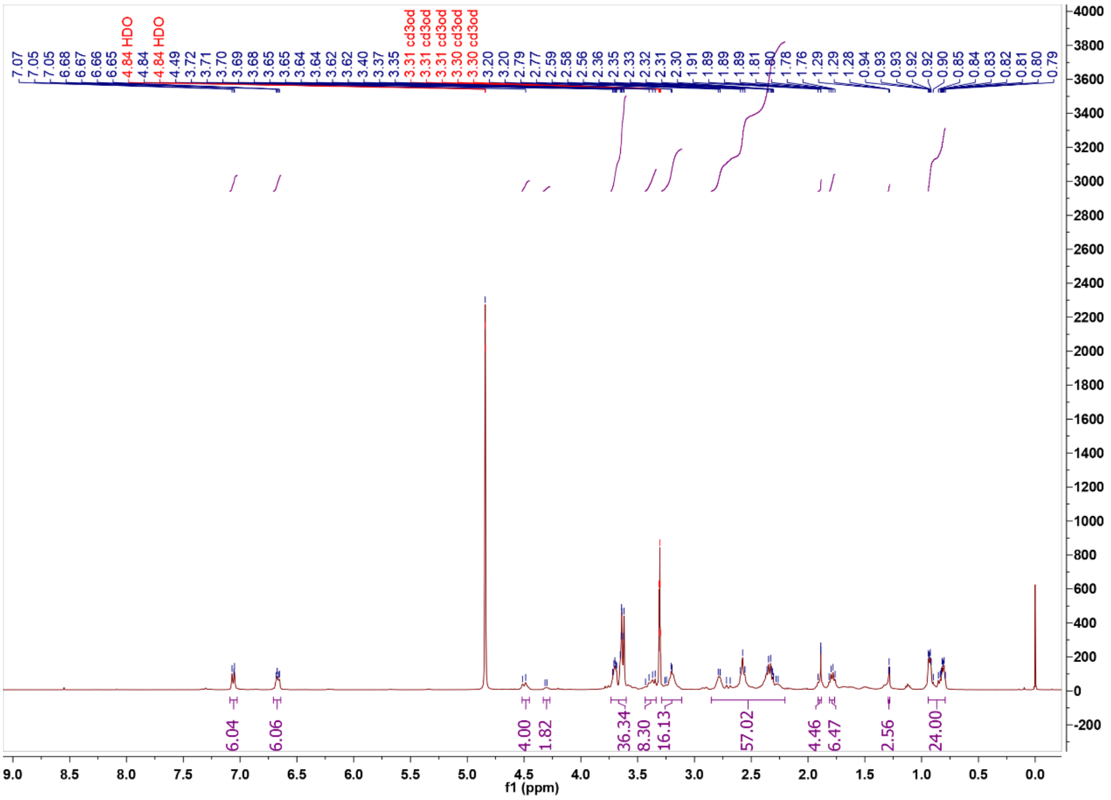


**Figure S13.** $^{1}H$ NMR spectrum of compound **3a.**

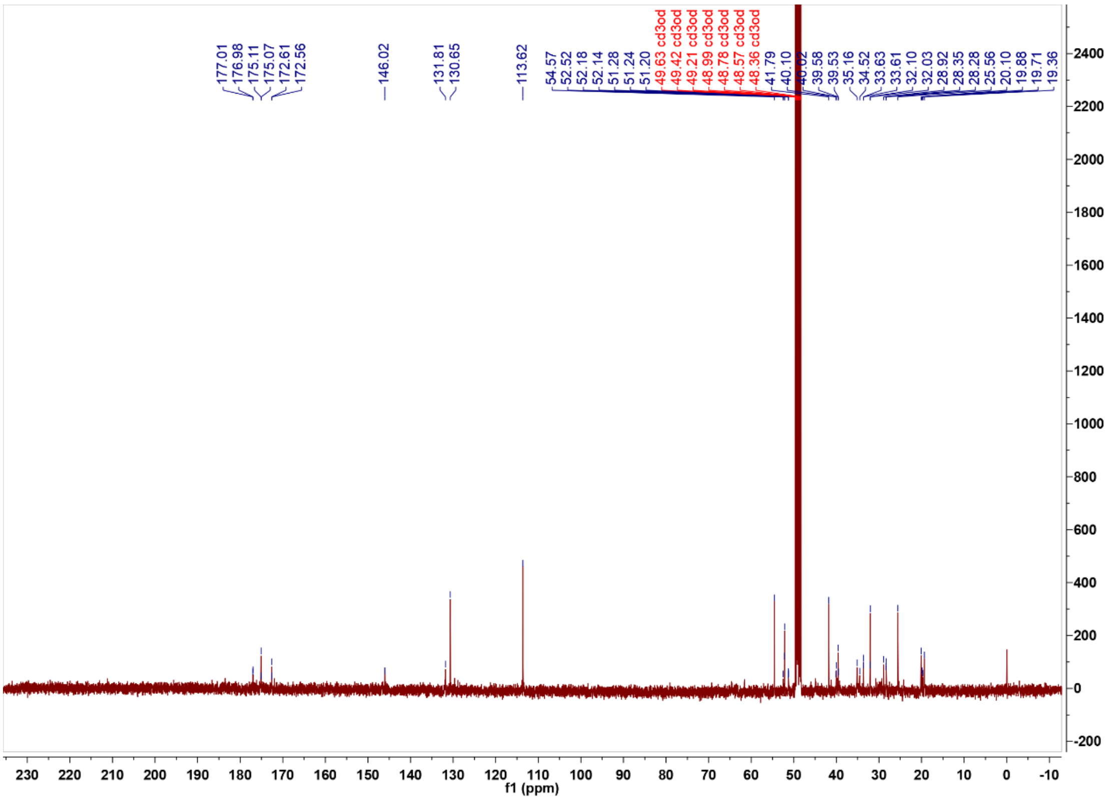


**Figure S14.** $^{13}C$ NMR spectrum of compound **3a.**

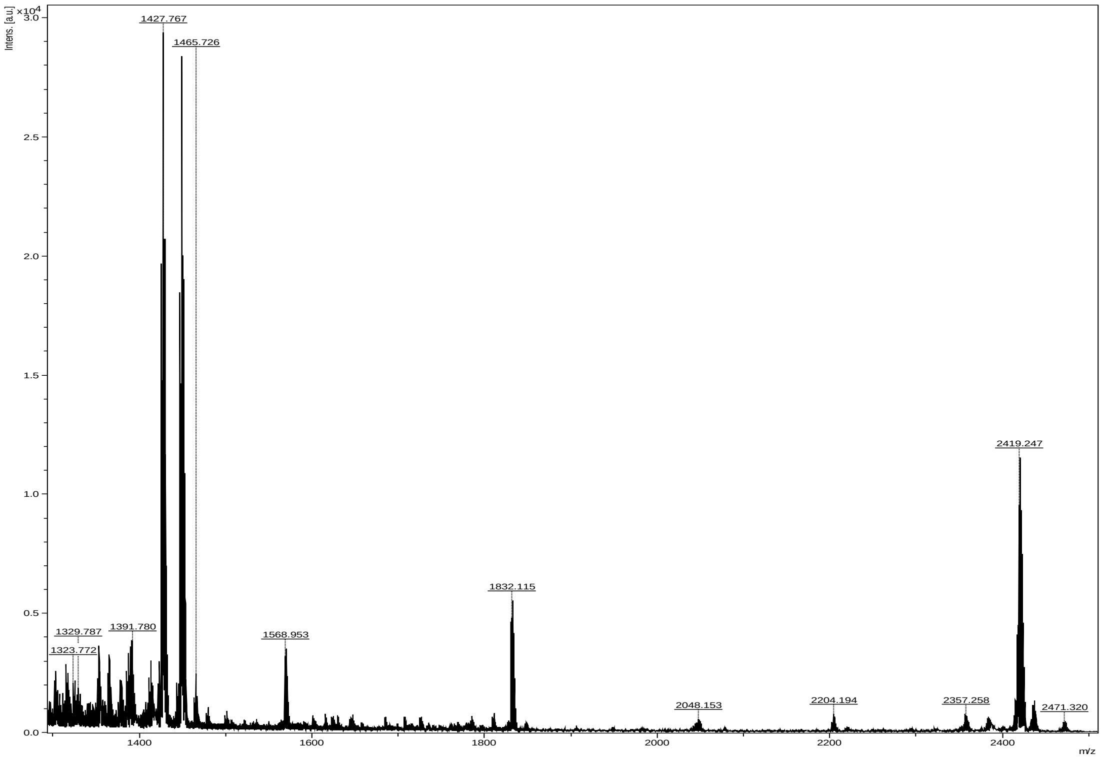


**Figure S15.** MALDI-TOF spectrum of compound **3a.**

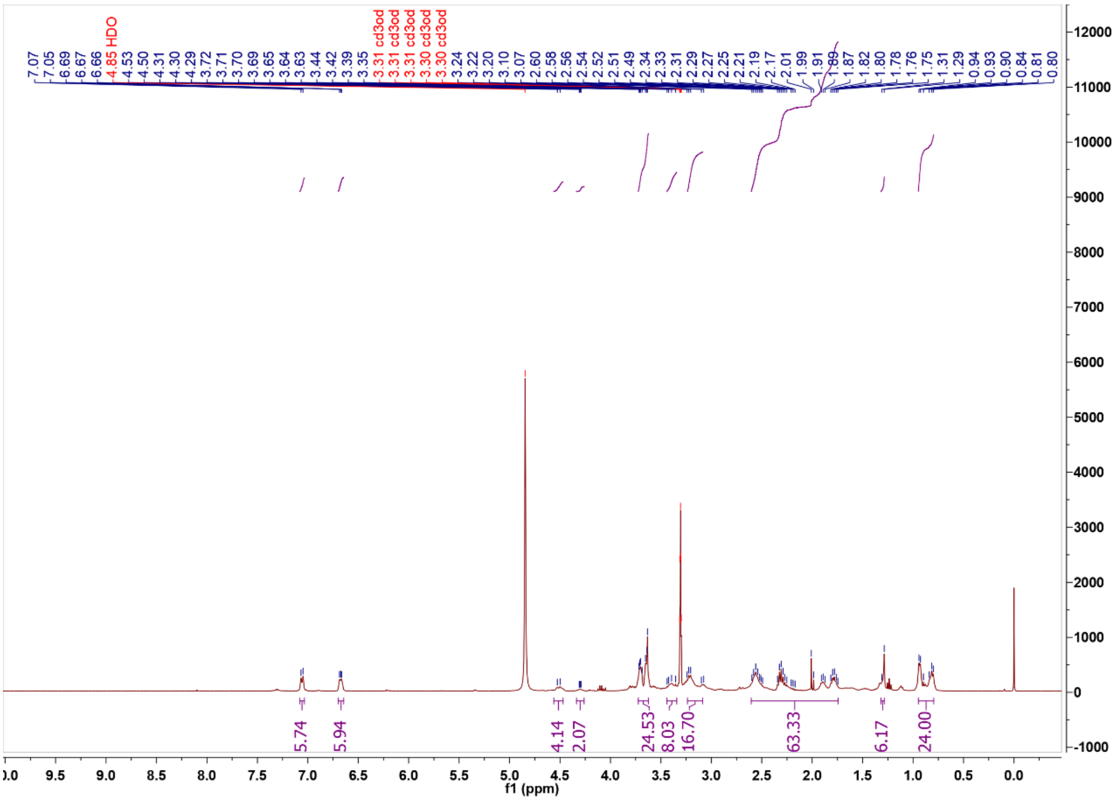


**Figure S16.** $^{1}$H NMR spectrum of compound **3b.**

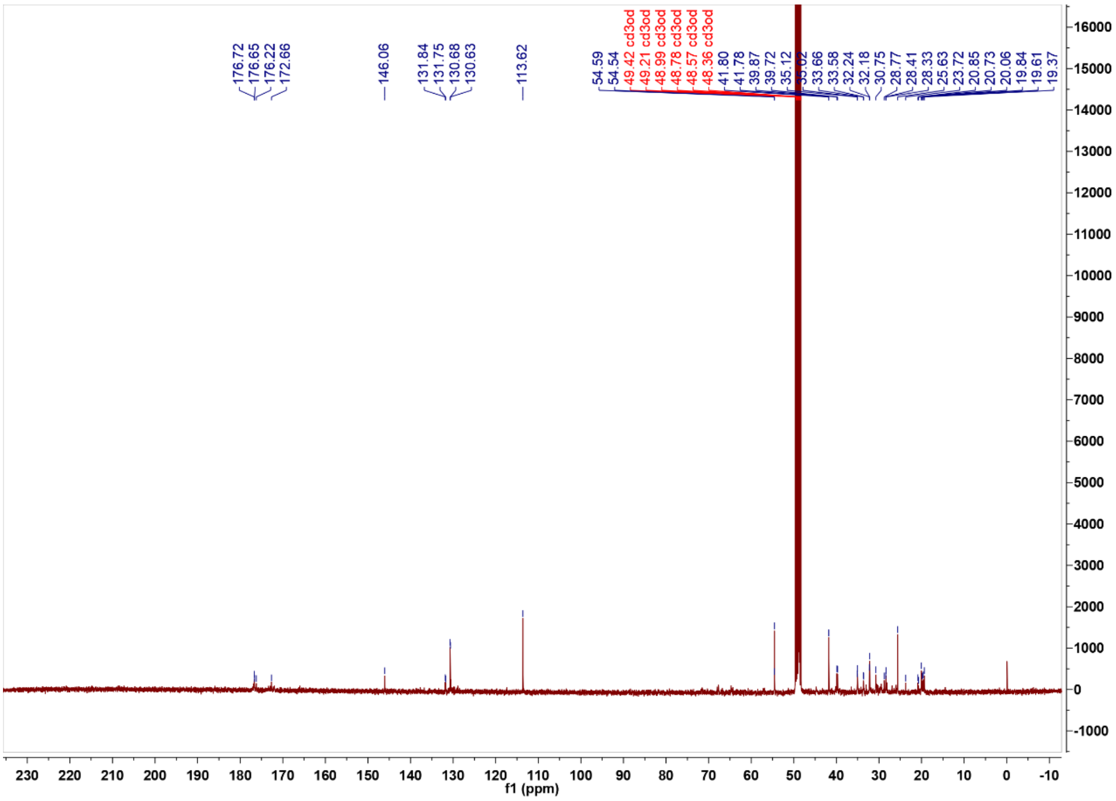


**Figure S17.** $^{13}C$ NMR spectrum of compound **3b.**

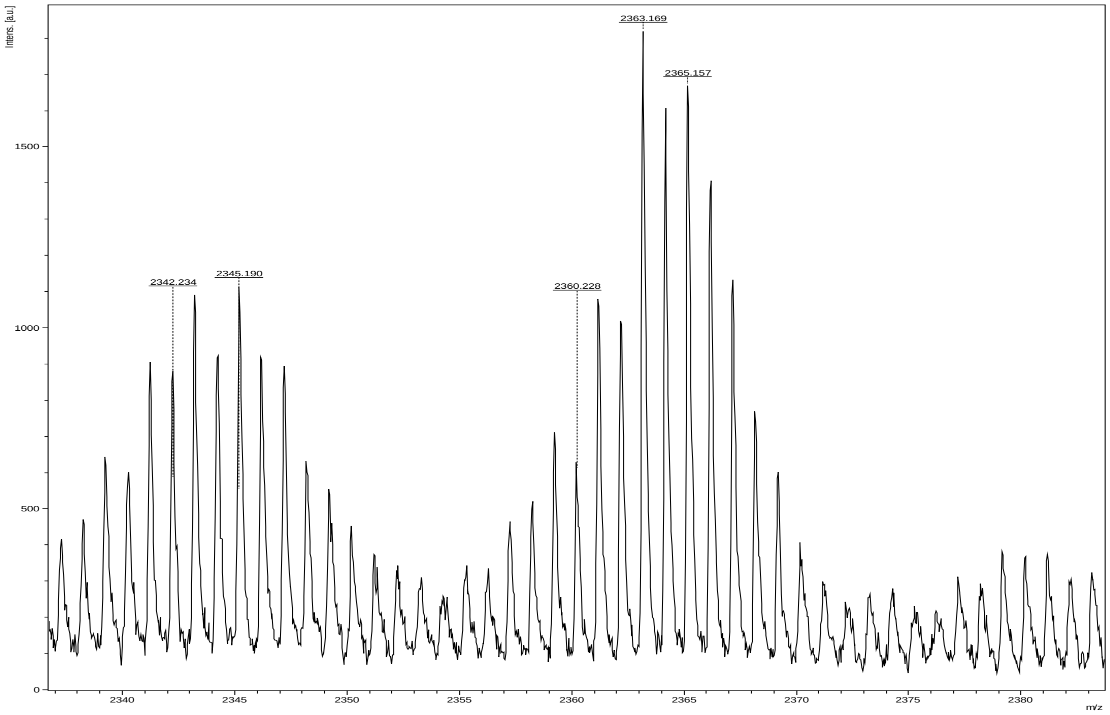


**Figure S18.** MALDI-TOF spectrum (expansion of *m*/*z* 2337–2384) of compound **3b.**

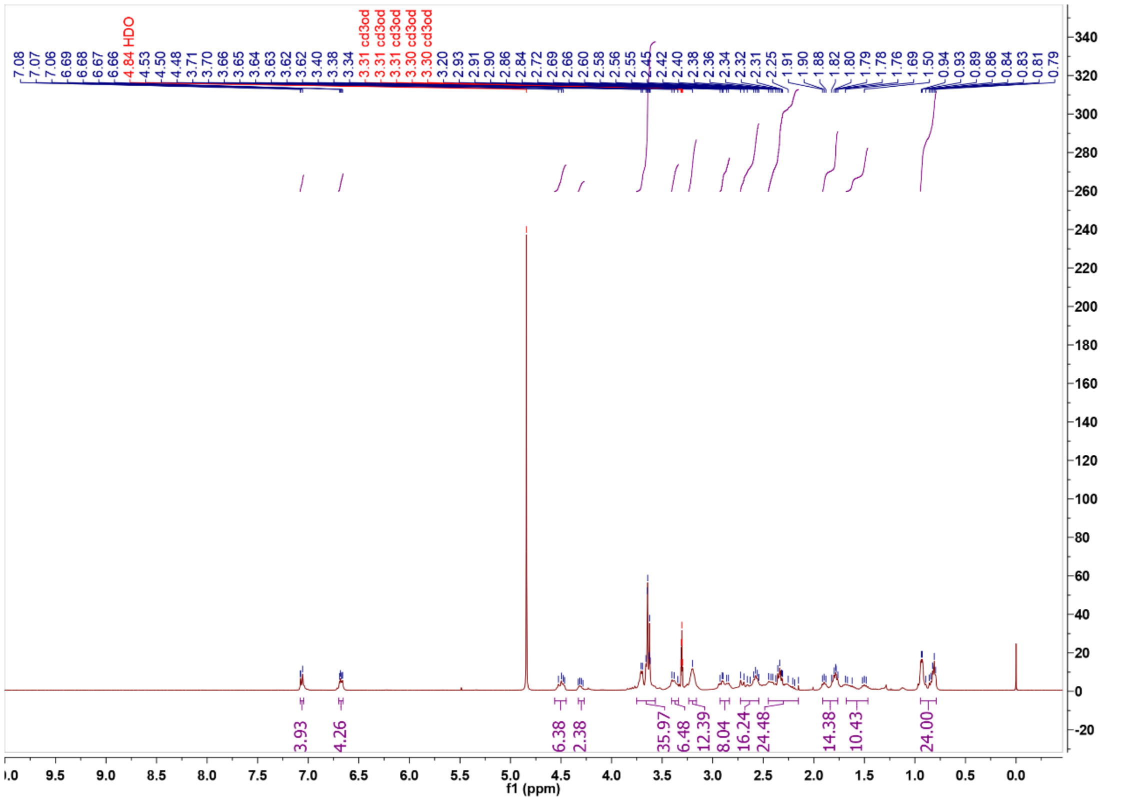

**Figure S19.** $^1$H NMR spectrum of compound **4a.**

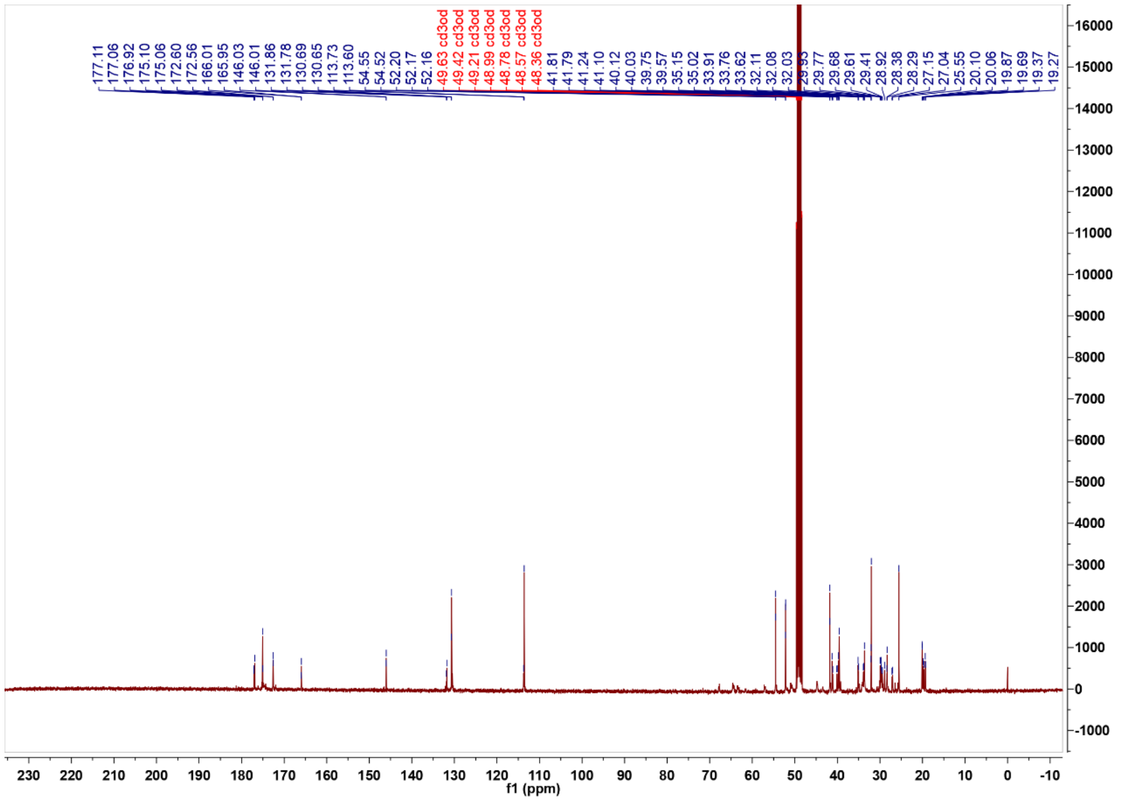

**Figure S20.** $^{13}$C NMR spectrum of compound **4a.**

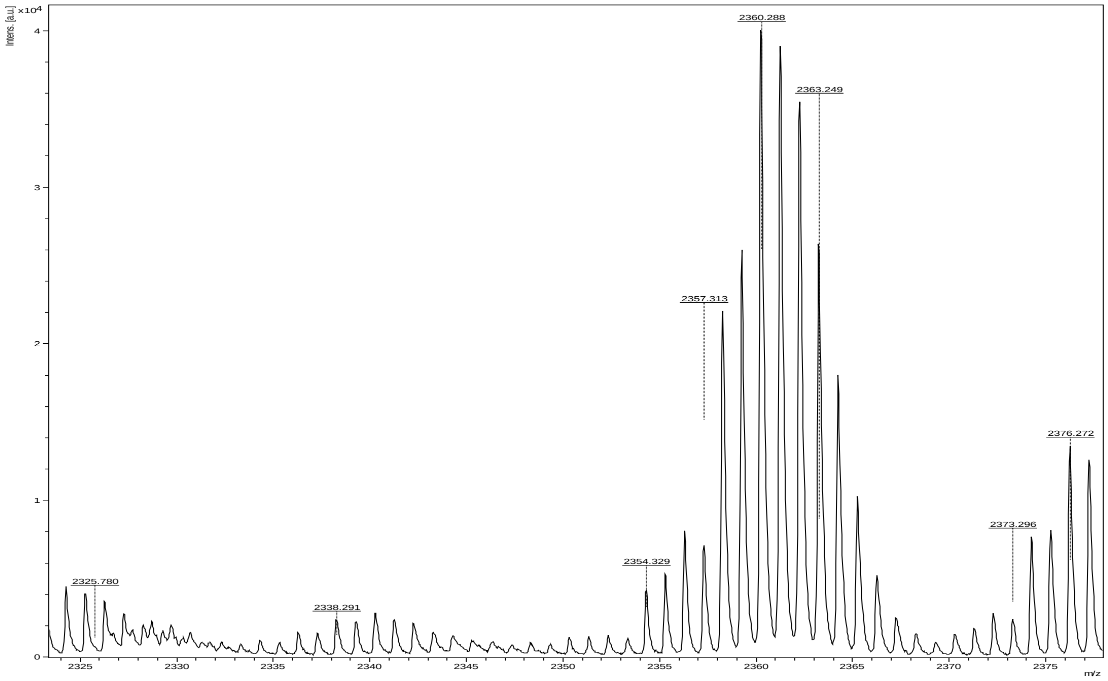


**Figure S21.** MALDI-TOF spectrum (expansion) of compound **4a.**

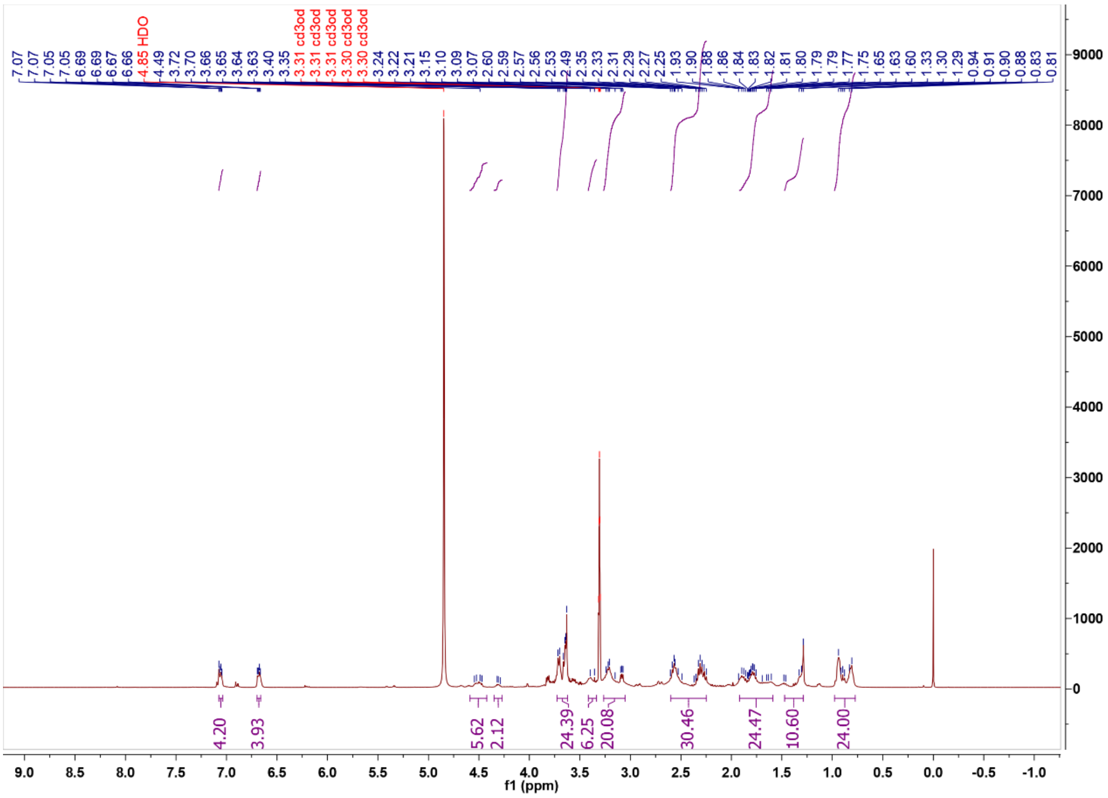


**Figure S22.** $^{1}$H NMR spectrum of compound **4b.**

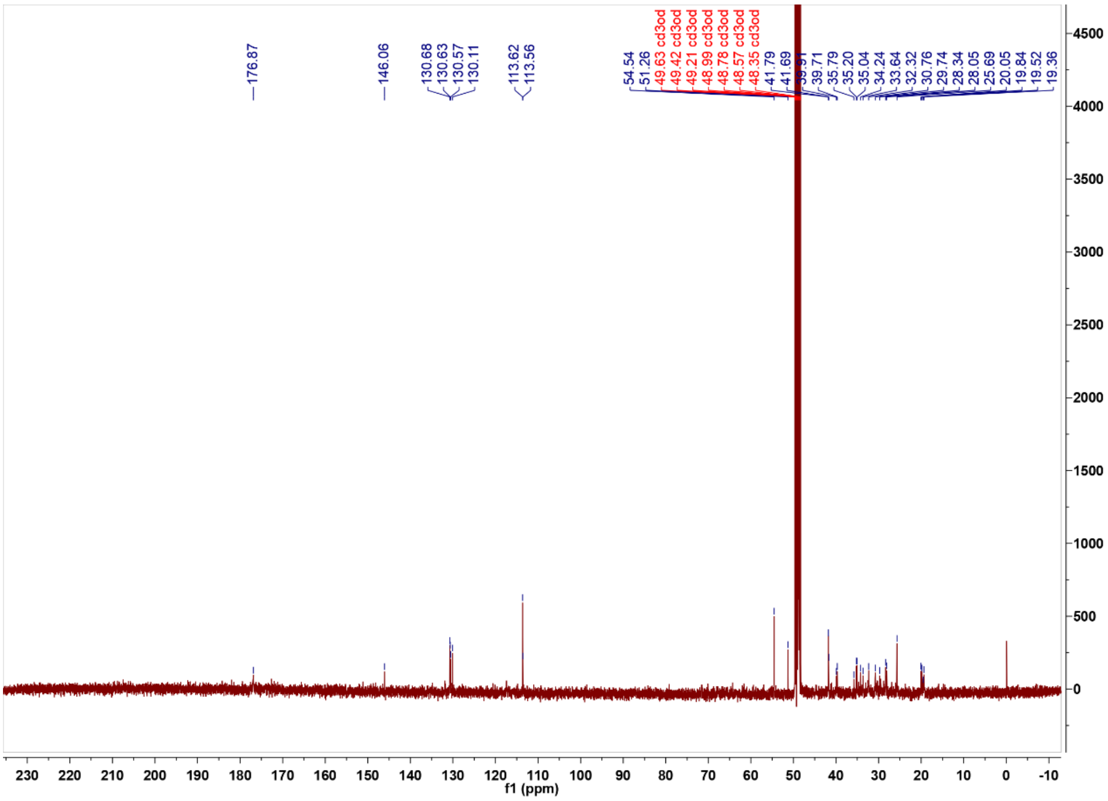


**Figure S23.** $^{13}$C NMR spectrum of compound **4b.**

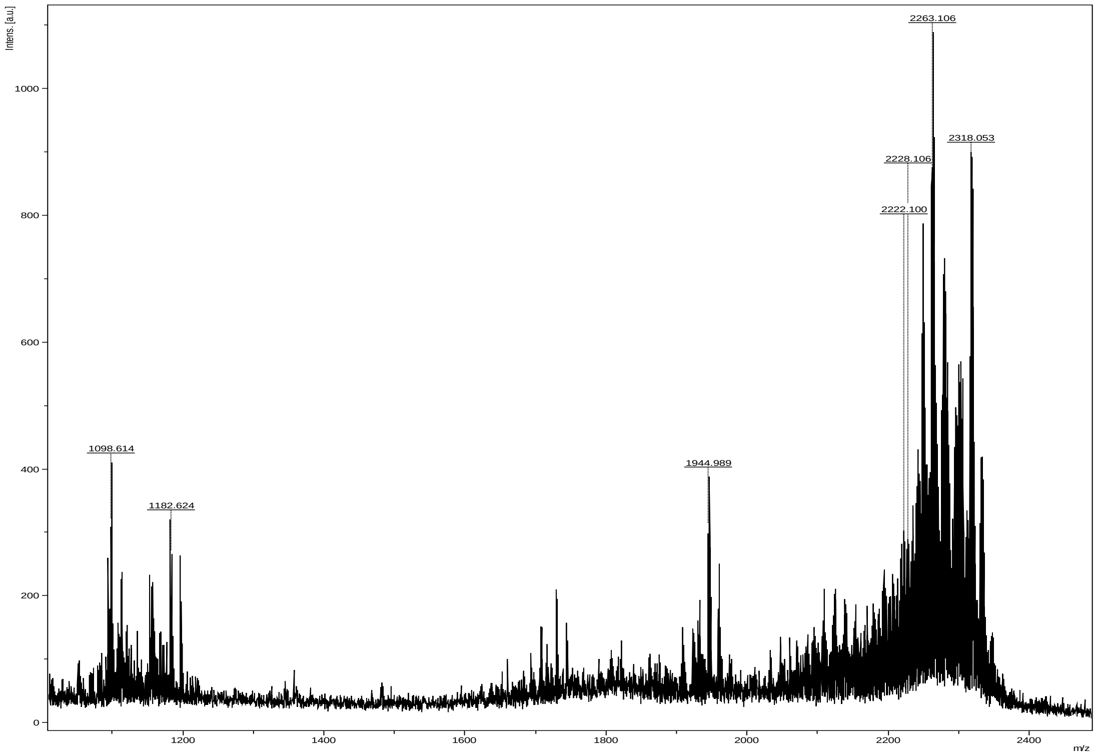


**Figure S24.** MALDI-TOF spectrum obtained for compound **4b.**

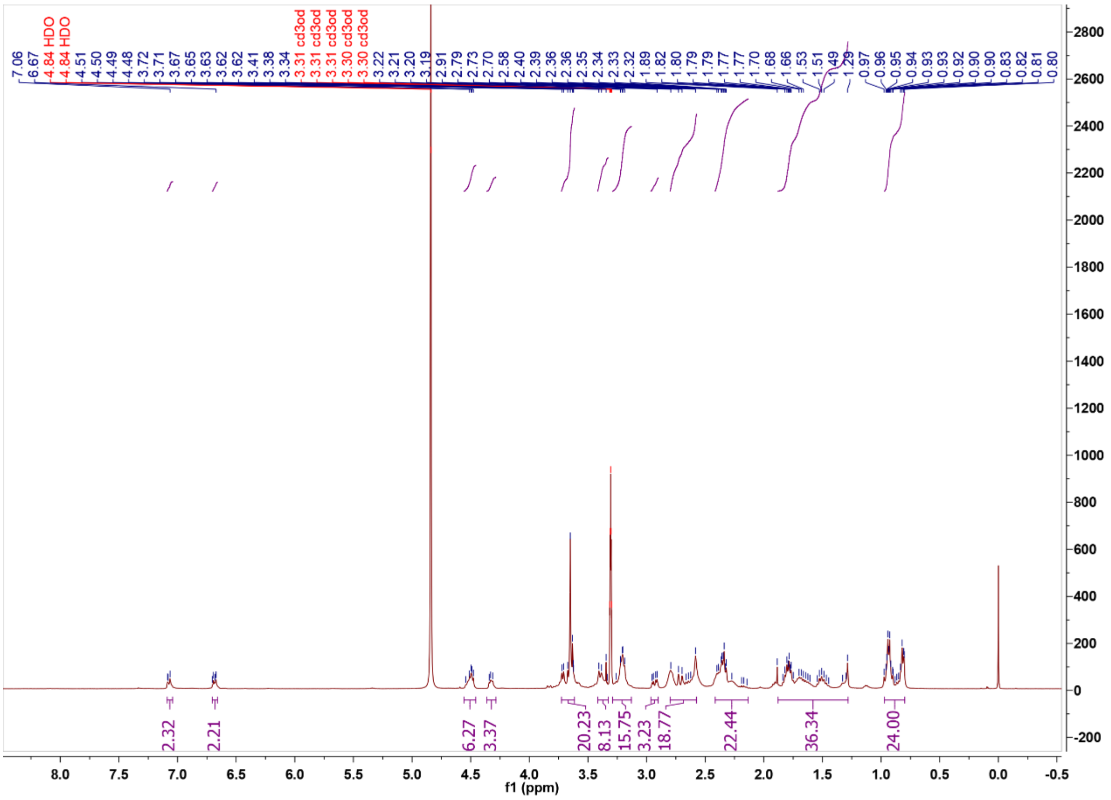

**Figure S25.** $^{1}H$ NMR spectrum of compound **5a.**

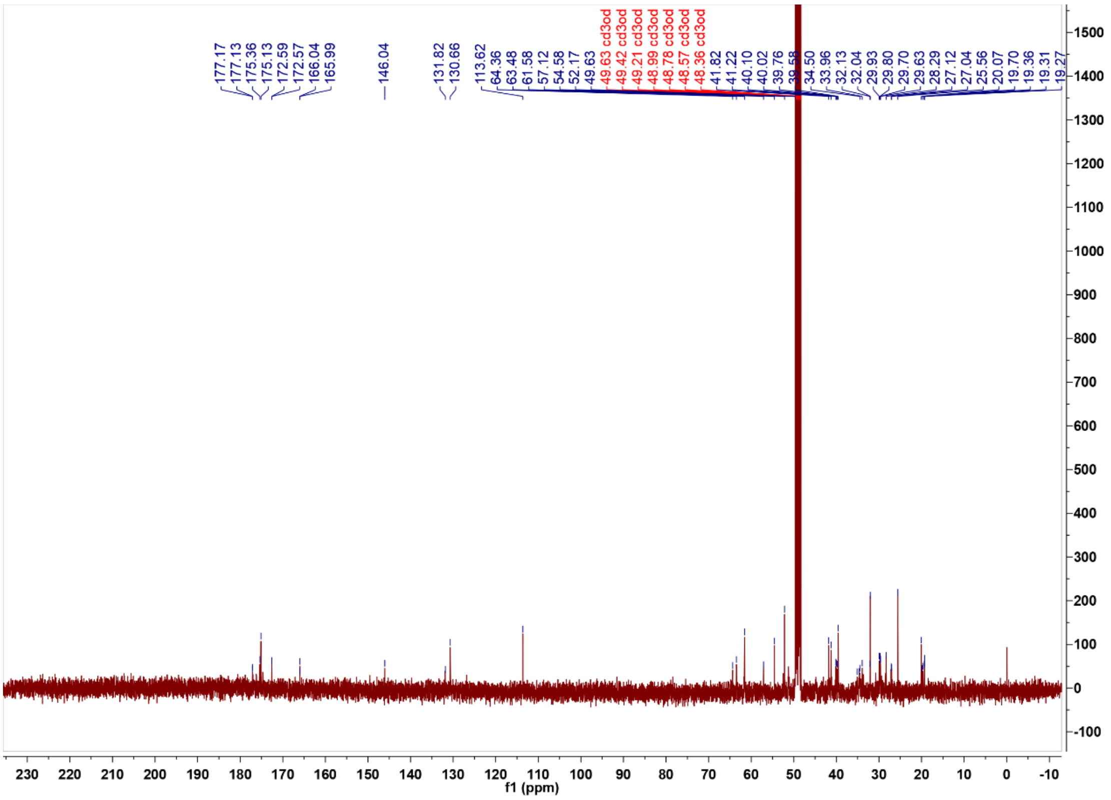

**Figure S26.** $^{13}C$ NMR spectrum of compound **5a.**

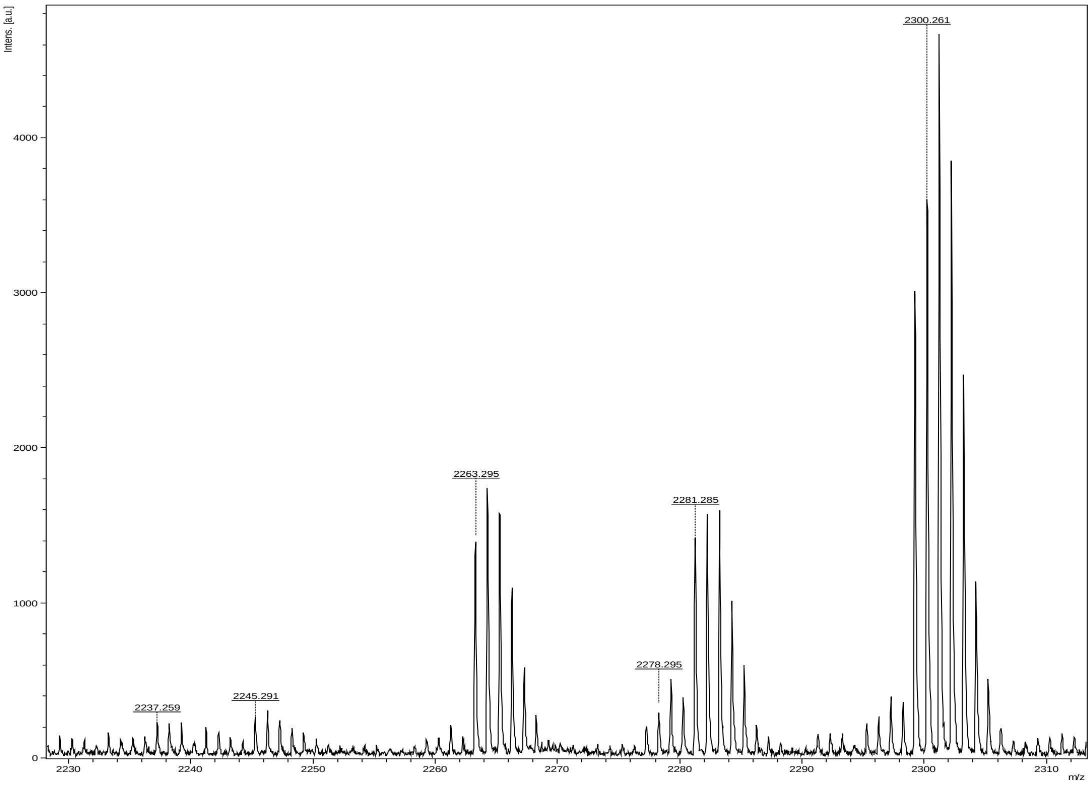


**Figure S27.** MALDI-TOF spectrum of compound **5a.**

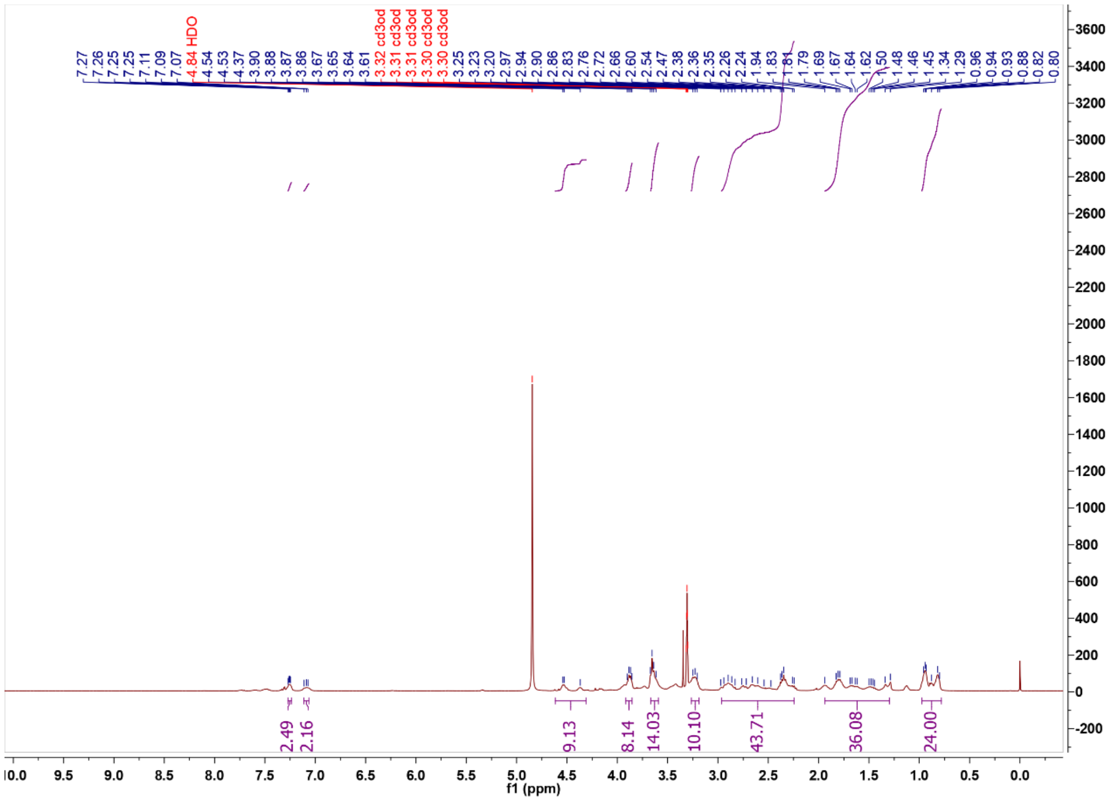


**Figure S28.** $^{1}$H NMR spectrum of compound **5b.**

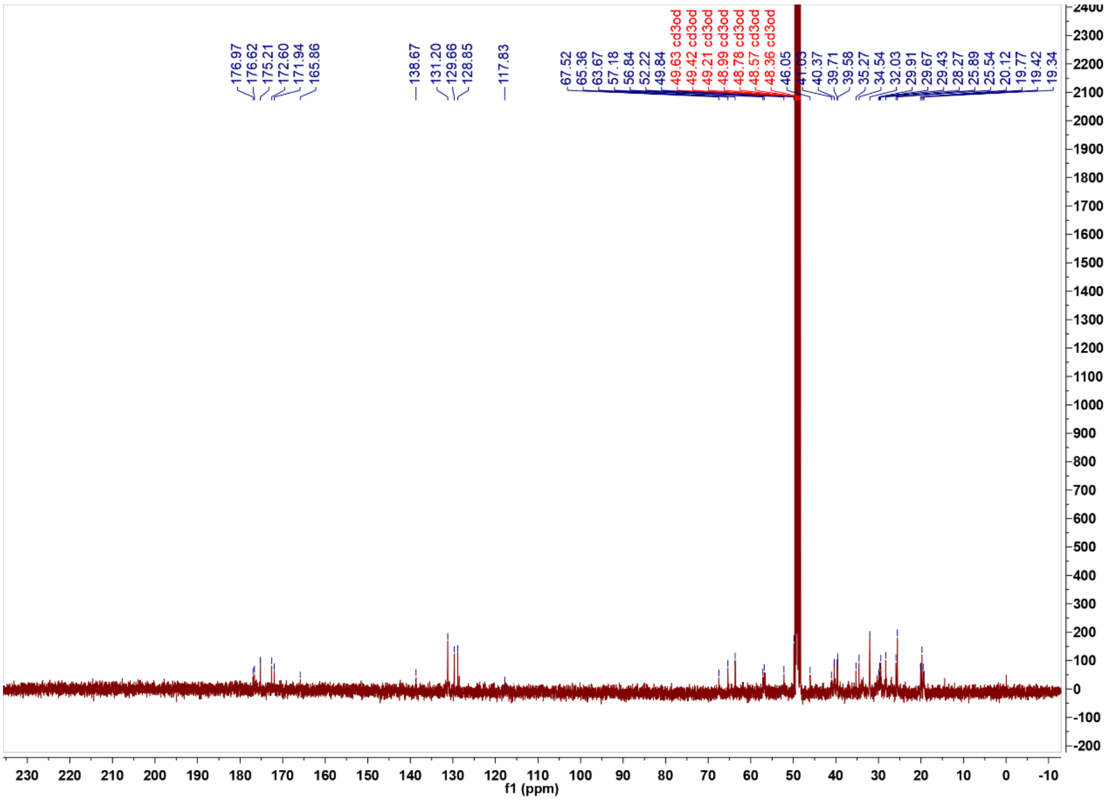


**Figure S29.** $^{13}C$ NMR spectrum of compound **5b.**

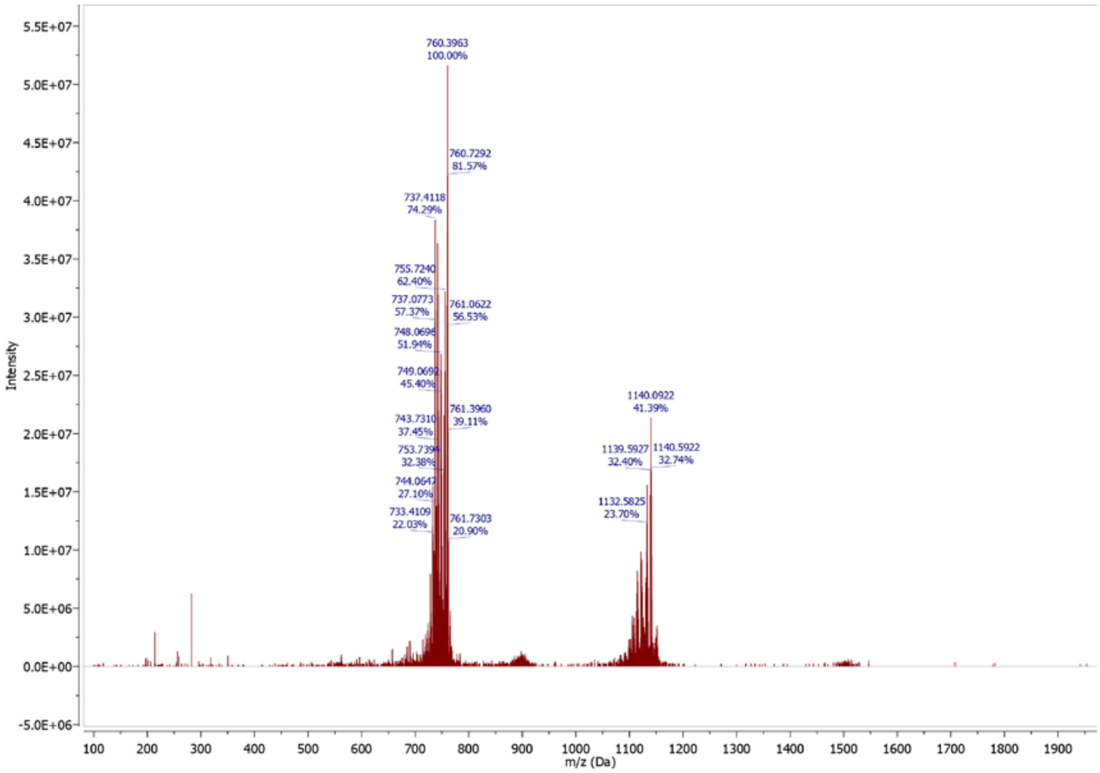


**Figure S30.** HRMS spectrum obtained for compound **5b.**

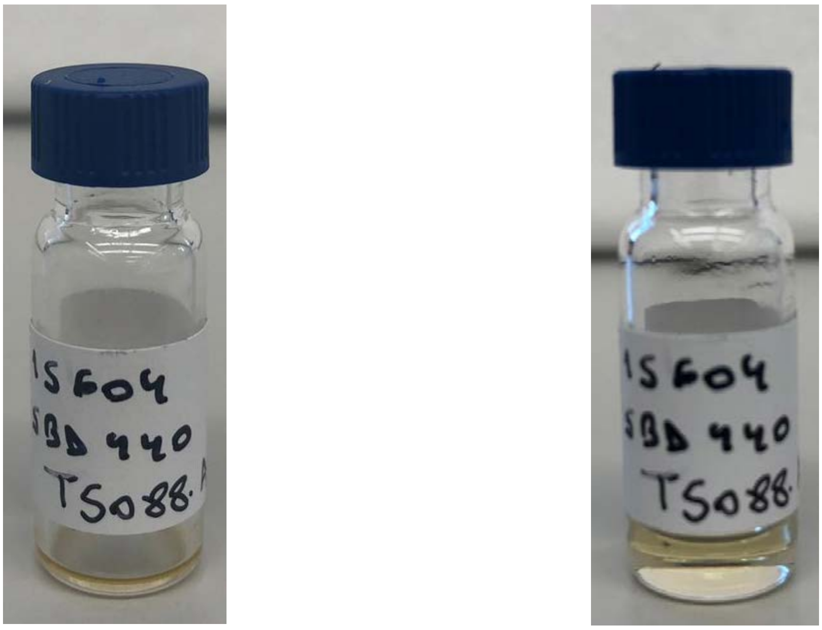

(**a**) (**b**)

**Figure S31**. Water solubility—compound **1b** before (**a**) and after dissolution in de-ionized water (**b**).

## 3. Evaluation of Anticancer Activity of the Dendrimers, Chlorambucil, and Biotin

PAMAM-$NH_2$ was tested according procedure described in 2.5.

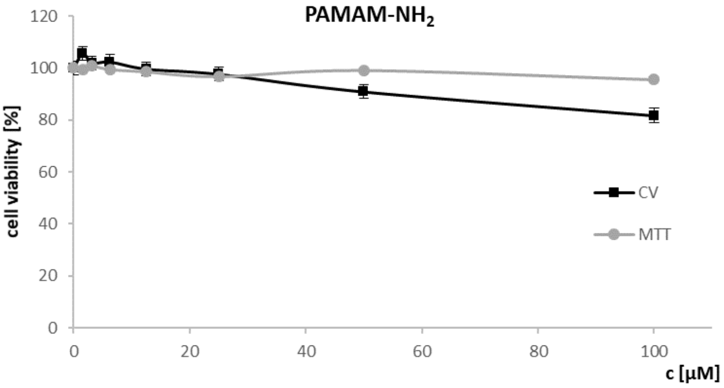


**Figure S32**. Dose-dependent response of PC-3 cells treated with PAMAM-$NH_2$, CV, and MTT assays (72 h).
